# Effects of acute caffeine intake on muscular power during resistance exercise: a systematic review and meta-analysis

**DOI:** 10.3389/fnut.2025.1686283

**Published:** 2025-10-07

**Authors:** Yuchun Xiao, Li Ding, Zhenbo Xu, Jue Liu, Li Guo, Matthew J. Barnes, Yinhang Cao, Olivier Girard

**Affiliations:** ^1^Physical Education Teaching and Research Department, Hunan Institute of Technology, Hengyang, China; ^2^School of Athletic Performance, Shanghai University of Sport, Shanghai, China; ^3^Department of Rehabilitation Medicine, Huashan Hospital, Fudan University, Shanghai, China; ^4^School of Exercise and Health, Shanghai University of Sport, Shanghai, China; ^5^School of Sport, Exercise and Nutrition, Massey University, Palmerston North, New Zealand; ^6^School of Human Sciences (Exercise and Sport Science), The University of Western Australia, Perth, WA, Australia

**Keywords:** caffeine supplementation, resistance exercise, movement velocity, power output, dose–response

## Abstract

**Background:**

This study examined the effects of caffeine on movement velocity and power output during resistance exercises and explored moderating factors influencing these effects.

**Methods:**

A systematic search of five databases was conducted through June 2025. A random-effects model was used to assess the effect of caffeine on muscular power-related variables, such as bar velocity and power output, during resistance exercises with a fixed number of repetitions. Subgroup analyses were performed based on sex, caffeine dose, habitual caffeine consumption, muscle group, and load.

**Results:**

Twelve studies comprising 230 participants were included. Caffeine significantly improved mean velocity (SMD = 0.42, 95% CI: 0.19–0.65, *p* < 0.05, *I^2^* = 85%) and mean power output (SMD = 0.21, 95% CI: 0.12–0.30, *p* < 0.05, *I^2^* = 14%) during resistance exercises. Greater improvements in mean velocity were observed in males (SMD: 0.56 vs. 0.22), and habitual caffeine consumption < 3 mg/kg/day (SMD: 0.87 vs. 0.21) (all *p* < 0.01 for subgroup comparisons). Furthermore, although caffeine increased mean velocity at all caffeine doses (SMD: 0.31–0.78), muscle groups (SMD: 0.32–0.54) and loads (SMD: 0.37–0.49) (all *p* < 0.01), no significant differences were observed between subgroups (all *p* > 0.01 for subgroup comparison).

**Conclusion:**

Caffeine ingestion enhances movement velocity and power output during resistance exercises, regardless of load. These benefits were more pronounced in males, at higher caffeine doses, among low habitual caffeine consumers, and during lower-body exercises.

**Systematic review registration:**

https://www.crd.york.ac.uk/PROSPERO/view/CRD42024616920.

## 1 Introduction

It is well established that sports such as track and field and team sports rely heavily on power-based actions like jumping and sprinting (1, 2). Resistance training remains a cornerstone of strength and conditioning programs aimed at developing muscular power (3). Recently, advanced methods including velocity-based training and blood flow restriction have emerged to optimize training outcomes (4, 5). Concurrently, sport supplements have attracted widespread attention as alternative approaches to boost muscular power during resistance exercise (6–8).

Caffeine (1,3,7-trimethylxanthine), classified as a nutritional ergogenic aid by the International Olympic Committee, has been demonstrated to improve muscular strength during resistance exercises (9). Meta-analyses report that caffeine doses of 1–7 mg/kg produce small improvements in maximal strength (one-repetition maximum [1RM]), with *trivial* effect sizes ranging from 0.17 to 0.20 (10, 11). However, maximal strength expression is less frequently prescribed in practical settings. Instead, resistance training often emphasizes muscular power at submaximal loads, aiming to improve the ability to lift heavy loads at high velocities, especially for athletes (12).

Recent reviews suggest that caffeine may have a greater effect on muscular power – specifically movement velocity and power output – than on maximal strength during resistance exercise (13, 14). However, there is limited meta-analytic research examining the effect of caffeine on muscular power during resistance exercise. To date, only one meta-analysis has examined the effects of caffeine supplement (1–9 mg/kg) on muscular power during resistance exercises (12). This study included 12 studies with 151 participants, reporting a significant increase in mean velocity (MV) during bench press and squat across loads from 10 to 100% of 1RM (12). However, the study had notable limitations (12): (a) inclusion of studies using multi-ingredient caffeinated supplements (e.g., coffee and energy drinks), making it difficult to isolate the independent effect of caffeine (15); and (b) lack of subgroup analyses by sex, dose, and habitual caffeine consumption (see next paragraph for details), which may moderate the ergogenic response (16). In light of several recent studies published (17–25) since 2020 (12), an updated meta-analysis is warranted.

Most meta-analysis have focussed on overall effects, without addressing key moderating factors (12, 26). However, individual and methodological variables are known to influence the ergogenic response to caffeine and warrant subgroup analysis. For instance, sex-related differences in caffeine metabolism and neuromuscular function may affect outcomes (16, 27); higher doses are generally associated with greater performance benefits (28); habitual caffeine intake can alter sensitivity to caffeine’s stimulatory effects (29); and upper- vs. lower-body exercises may differ in responsiveness due to variations in muscle mass and recruitment patterns (30). These considerations provide a strong rationale for examining subgroup effects across sex, dose, habitual intake, and muscle group.

The aim of this study was to examine the effects of caffeine on muscular power during resistance exercises and to explore influencing factors to inform practical recommendations for exercisers.

## 2 Materials and methods

This meta-analysis followed the Preferred Reporting Items for Systematic Reviews and Meta-Analyses (PRISMA) guidelines (31) and was registered with the International Systematic Review Prospective Register (PROSPERO) (CRD42024616920).

### 2.1 Literature search

A comprehensive search was conducted in PubMed, Web of Science, EMBASE and CNKI (China National Knowledge Infrastructure) databases from inception to 10 June 2025. Search terms included combinations of the following keywords using Boolean operators (“AND,” “OR,” “NOT”): *caffeine, caffeinated, resistance exercise, resistance training, strength training, power, velocity, speed, performance,* and *exercises*. To ensure no relevant literature was missed, a manual search was also performed via Google Scholar.

### 2.2 Selection criteria

Studies were included based on PICOS criteria (participants, interventions, comparators, outcomes, and study design): (1) healthy adult participants; (2) caffeine administered alone, without combination with other ergogenic substances such as energy drinks or chocolate; (3) use of a placebo comparator; (4) outcomes including MV and mean power output (MPO) during resistance exercises with a fixed number of repetitions; and (5) single- or double-blind crossover design. Studies not meeting these criteria were excluded. Furthermore, this review did not include any preprints or unpublished research, as these data have not undergone peer review. The selection process was conducted by two independent investigators (B.X. and L.D.), with discrepancies resolved by a third investigator (Y.C.).

### 2.3 Study coding and data extraction

The following data were extracted from each study: (1) study design; (2) participant characteristics (e.g., sample size, age, training status, habitual caffeine consumption, and sex); (3) caffeine intake strategy (e.g., dose, form of administration, timing of ingestion, and caffeine withdrawal); (4) exercise protocol (type of exercise and load); and (5) main findings.

### 2.4 Assessment of methodological quality

Methodological quality and risk of bias were assessed in accordance with Cochrane guidelines (32). Two independent investigators evaluated each study using the Cochrane Risk of Bias Assessment Tool using Review Manager 5.4 software (Copenhagen: The Nordic Cochrane Center, The Cochrane Collaboration, 2014). The assessment included the following domains: (1) random sequence generation; (2) allocation concealment; (3) blinding of participants and personnel; (4) blinding of outcome assessment; (5) incomplete outcome data; (6) selective reporting; and (7) other potential biases. Each domain was rated as “low risk” (“+”), “some concerns” (“-”), or “unclear risk” (“×”).

### 2.5 Statistical analyses

This meta-analysis was conducted using STATA 14 (Stata Corp., College Station, TX, United States) and R software. A random-effects model was applied to estimate differences in MV and MPO between caffeine and placebo groups, based on the mean values, standard deviations, and correlations. Results were reported as standardized mean differences (SMD) (Hedge’s g) with 95% confidence intervals (CI), with significance set at *p* < 0.05. Since no study included in our review reported correlation values, a conservative correlation of 0.5 was assumed for all studies (12). For studies reporting multiple muscular power outcomes under different conditions (i.e., varying caffeine dosages and loads), SMDs and variances were calculated separately for each outcome, with average SMD and variance values used for analysis (12). The magnitude of SMD was interpreted as: (a) *trivial* (SMD < 0.20); (b) *small* (0.20 ≤ SMD < 0.50); (c) *moderate* (0.50 ≤ SMD < 0.80); and (d) *large* (SMD ≥ 0.80) (32). Study heterogeneity was calculated using the *I^2^* statistic, classified as *low* (*I^2^* < 25%), *moderate* (25% ≤ *I^2^* ≤ 50%), or *high* (*I^2^* > 50%) (33). Publication bias was assessed using funnel plots and Egger’s test. In the sensitivity analysis, the pooled results were examined by sequentially excluding each included study.

Subgroup analyses were conducted to examine the effects of caffeine on muscular power during resistance exercise based on the following factors: (1) sex (male and female); (2) caffeine dose (low [≤ 3 mg/kg], moderate [> 3 mg/kg to ≤ 6.0 mg/kg], and high [> 6 mg/kg]) (16); (3) habitual caffeine consumption (naive-to-mild [< 3.0 mg/kg/day] and moderate-to-high [≥ 3.0 mg/kg/day]) (34); and (4) muscle group (upper and lower body); (5) load (low [< 30% 1RM], moderate [30–70% 1RM], and high [> 70% 1RM]) (35, 36). Additionally, given the multiple subgroup analyses (five groups), the Bonferroni method was applied, adjusting the significance threshold for subgroup differences to *p* < 0.01 to reduce the risk of false positives.

## 3 Results

### 3.1 Study characteristics

**Figure 1:**
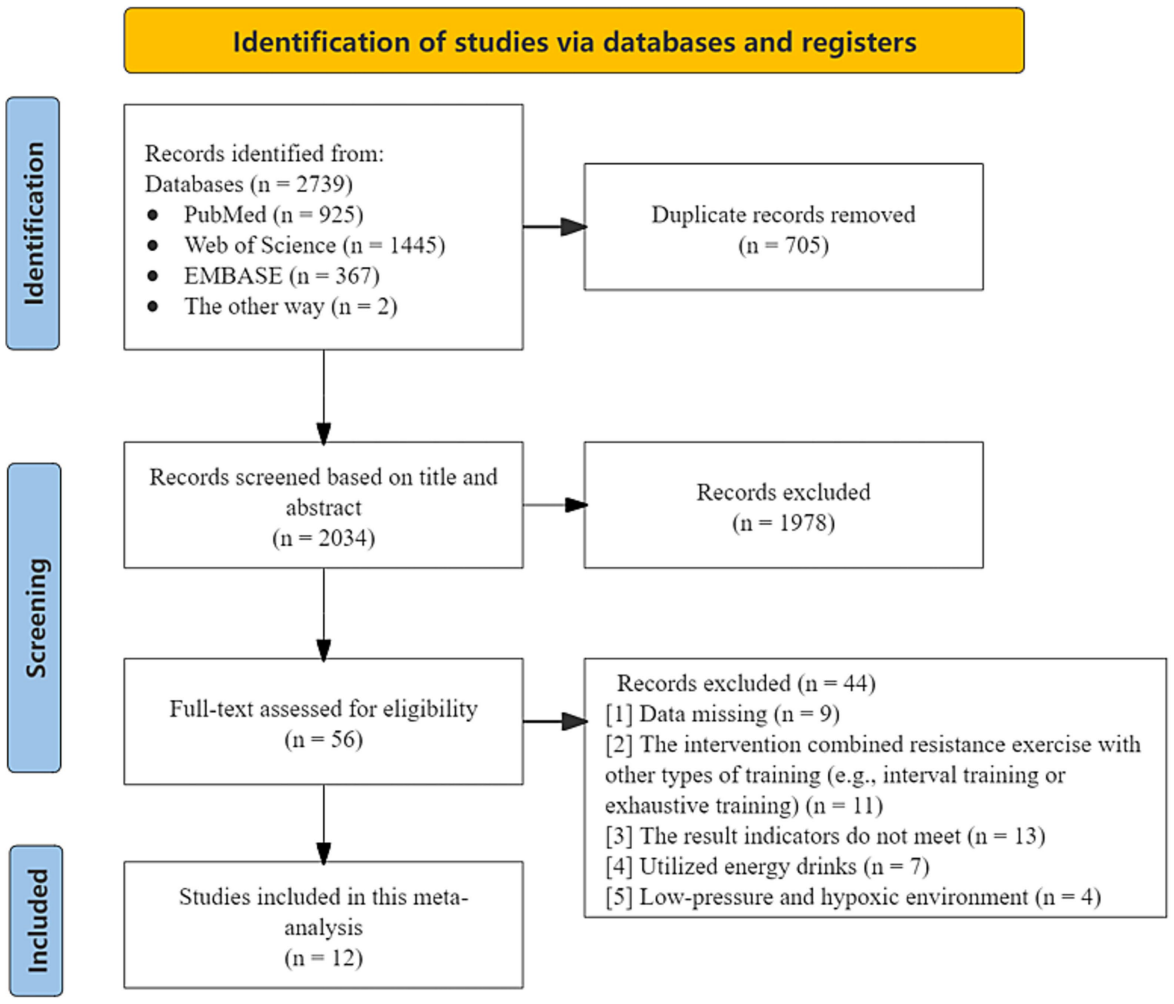
PRISMA flow diagram showing study selection.

A total of 2,739 studies were initially identified through database searches. After removing 705 duplicates, 2,034 studies were screened based on titles and abstracts. Fifty-six studies underwent full-text screening, and 12 studies met the inclusion criteria and were included in the meta-analysis (17–25, 28, 37, 38) (Figure 1). Due to multiple conditions within some studies (e.g., different sexes, doses or loads), several contributed more than one dataset, resulting in 67 trials from 12 studies in subgroup analysis (Table 1) (17–25, 28, 37, 38). The total sample size consisted of 230 resistance-trained or recreationally active participants, aged 20–29 years. The habitual caffeine consumption of most study participants ranged from naive to moderate (0–6 mg/kg/day), with the exception of one study reporting an intake of 632 mg/day. Reported caffeine doses varied from 3 to 12 mg/kg. All studies administered caffeine in liquid or capsule form 60 min before exercise, expect one, which used caffeinated gum 15 min prior.

**Table 1:** Characteristics of the included studies.

Study	Sample + age (yrs) + level	Habitual caffeine intake	Caffeine withdrawal (days)	Blinding	Caffeine form + dose (mg/kg) + timing (min)	Comparator	Exercise protocol	Outcomes
Montalvo-Alonso et al., 2024 (17)	38 M/38 F; 26.5 ± 8.5; resistance-trained	5.6 ± 4.25 mg/kg/day	3	Triple blind	Liquid; 6; 60	Placebo (maltodextrin)	Bench press and back squat in 25, 50, 75 and 90% 1RM	↑ MV (25% 1RM, F); MPO (50–75% 1RM, F) in bench press ↑ MV (50–90% 1RM, F and M); MPO (50–90% 1RM, F and M); in back squat → MPO (25 and 90% 1RM, F); MV (50 and 90% 1RM, F); MV (25–90% 1RM, M); MPO (25–90% 1RM, M) in bench press → MV (25% 1RM, F and M); MPO (25% 1RM, F and M) in back squat
Krawczyk et al., 2022 (19)	6 M/4 F; 26.4 ± 5.3/ 20.8 ± 1.5; resistance-trained	2.6 ± 2.2 mg/kg/day	3	Double blind	Caffeine; 3 and 6; 60	Placebo (N.A.)	Bench press in 50% 1RM	↑ MV (3 mg/kg); → MV (6 mg/kg)
Filip-Stachnik et al., 2022 (20)	12 F; 23.3 ± 0.8; recreationally active	5.7 ± 2.0 mg/kg/day	3–7	Double blind	Capsules; 3 and 6; 60	Placebo (flour)	Bench press in 50% 1RM	↑ MV (3 and 6 mg/kg)
Filip-Stachnik et al., 2021 (21)	12 M; 25.2 ± 1.3; resistance-trained	5.3 ± 1.4 mg/kg/day	1	Double blind	Capsules; 9 and 12; 60	Placebo (flour)	Bench press throw in 30% 1RM	↑ MV (9 and 12 mg/kg)
Filip-Stachnik et al., 2021 (22)	13 M; 21.9 ± 1.2; recreationally active	1.6 ± 0.6 mg/kg/day	7	Double blind	Capsules; 3 and 6; 60	Placebo (flour)	Bench press in 70% 1RM	↑ MV; MPO
Wilk et al., 2020 (23)	12 M; 25.3 ± 1.7; resistance-trained	4–6 mg/kg/day	7	Double blind	Capsules; 3 and 6; 60	Placebo (flour)	Bench press throw in 30% 1RM	↑ MV (3 and 6 mg/kg); MPO (3 and 6 mg/kg); MV (6 mg/kg)
Giraldez-Costas et al., 2020 (24)	9 M/3 F; 29 ± 8; recreationally active	<100 mg/day	5	Double blind	Capsules; 3; 60	Placebo (inert substance)	Bench press in 70% 1RM	↑ MV, MPO
Venier et al., 2019 (37)	19 M; 24 ± 5; recreationally active	67 ± 85 mg/day	3–6	Double blind	Gum; 3; 15	Placebo (caffeine-free gum)	Bench press in 50, 75 and 90% 1RM	↑ MV (50–90% 1RM)
Wilk et al., 2019 (25)	19 M; 26.8 ± 6.2; resistance-trained	5.2 ± 1.2 mg/kg/day	7	Double blind	Capsules; 3, 6, and 9; 60	Placebo (flour)	Bench press in 50% 1RM	↑ MPO (6 and 9 mg/kg); → MV (3, 6 and 9 mg/kg); MPO (3 mg/kg)
Ruiz-Fernández et al., 2023 (18)	10 M/10 F; 22.9 ± 3.6; resistance-trained	632 ± 490 mg/day	3	Double blind	Liquid; 3; 60	Placebo (maltodextrin)	Bench press and back squat in 25, 50, 75 and 90% 1RM	↑ MV (75–90% 1RM); MPO (75–90% 1RM) in bench press ↑ MV (25–90%1RM); MPO (75–90% 1RM) in back squat → MV (25–50% 1RM); MPO (25–50% 1RM) in bench press →MPO (25–50% 1RM) in back squat
Pallares et al., 2013 (28)	13 M; 21.9 ± 2.9; resistance-trained	≤70 mg/kg	2	Double blind	Capsules; 3, 6, and 9; 60	Placebo (dextrose)	Bench press and back squat in 25, 50, 75 and 90% 1RM	↑ MV (3, 6 and 9 mg/kg, 25–50% 1RM); MPO (3, 6 and 9 mg/kg, 25–50% 1RM); MV (6, 9 mg/kg, 75% 1RM); MV (9 mg/kg, 90% 1RM) in bench press ↑ MV (3, 6 and 9 mg/kg, 25–75% 1RM); MV (6, 9 mg/kg, 90% 1RM) in back squat → MV (3 mg/kg, 75–90% 1RM); MV (6 mg/kg, 90% 1RM); MPO (3, 6 and 9 mg/kg, 75–90% 1RM) in bench press → MV (3 mg/kg, 90% 1RM); MPO (3, 6 and 9 mg/kg, 25–90% 1RM) in back squat
Mora-Rodríguez et al., 2012 (38)	12 M; 19.7 ± 2.8; resistance-trained	≤60 mg/kg	1–1.5	Double blind	Capsules; 3; 60	Placebo (dextrose)	75% 1RM and loads that elicited a velocity of 1 m·s^−1^ in bench press and back squat	↑ MV in bench press and back squat

↑, increase; →, no difference; BP, bench press; F, females; M, males; MPO, mean power output; MV, mean velocity; N.A., not available; 1RM, one repetition maximum; SQ, squat.

### 3.2 Quality of study methods

**Figure 2:**
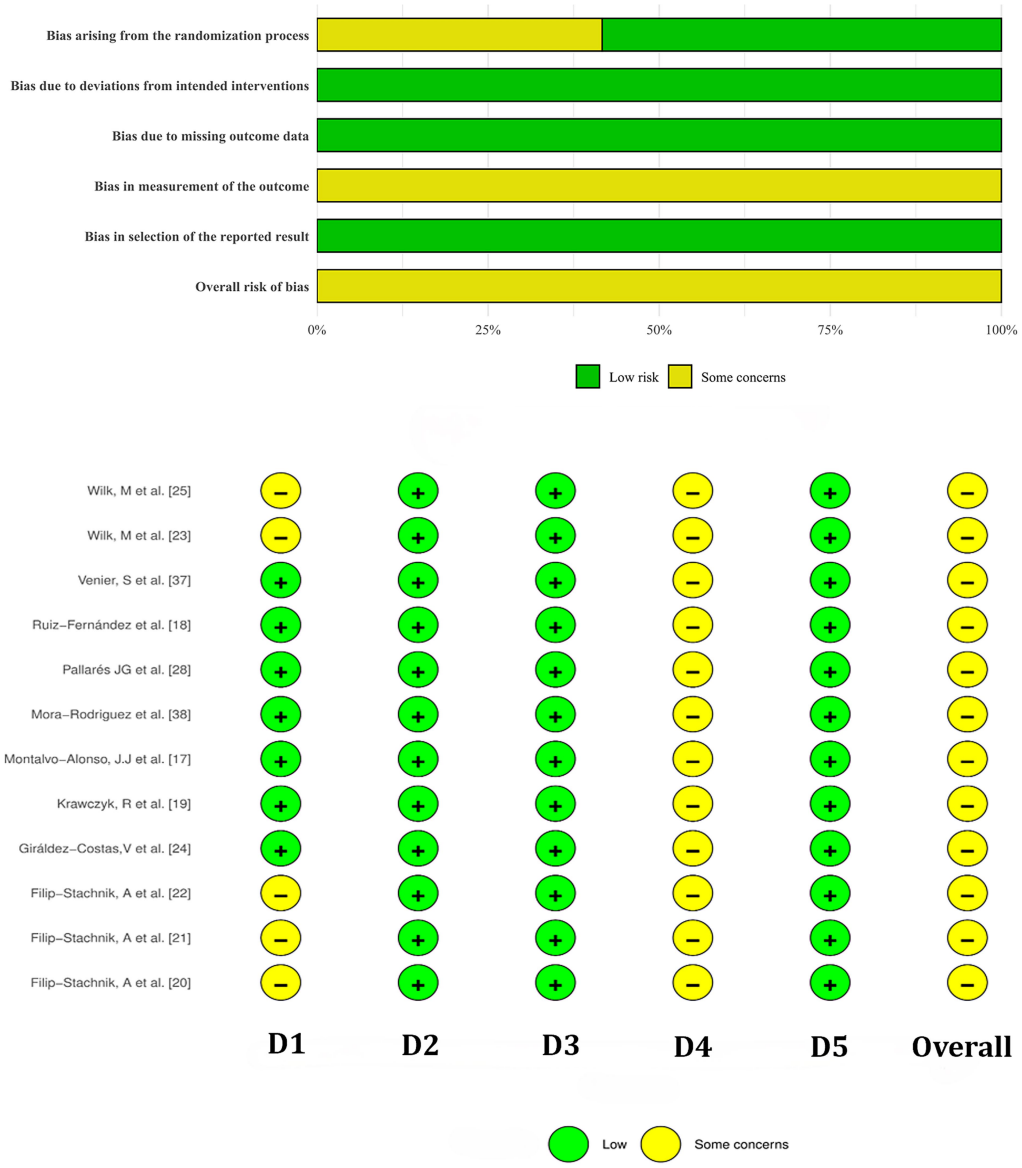
Risk of bias summary of included studies. D1, Bias arising from the randomization process; D2, Bias due to deviations from intended intervention; D3, Bias due to missing outcome data; D4, Bias in outcome measurement. D5: Bias in selection of the reported result.

**Figure 3:**
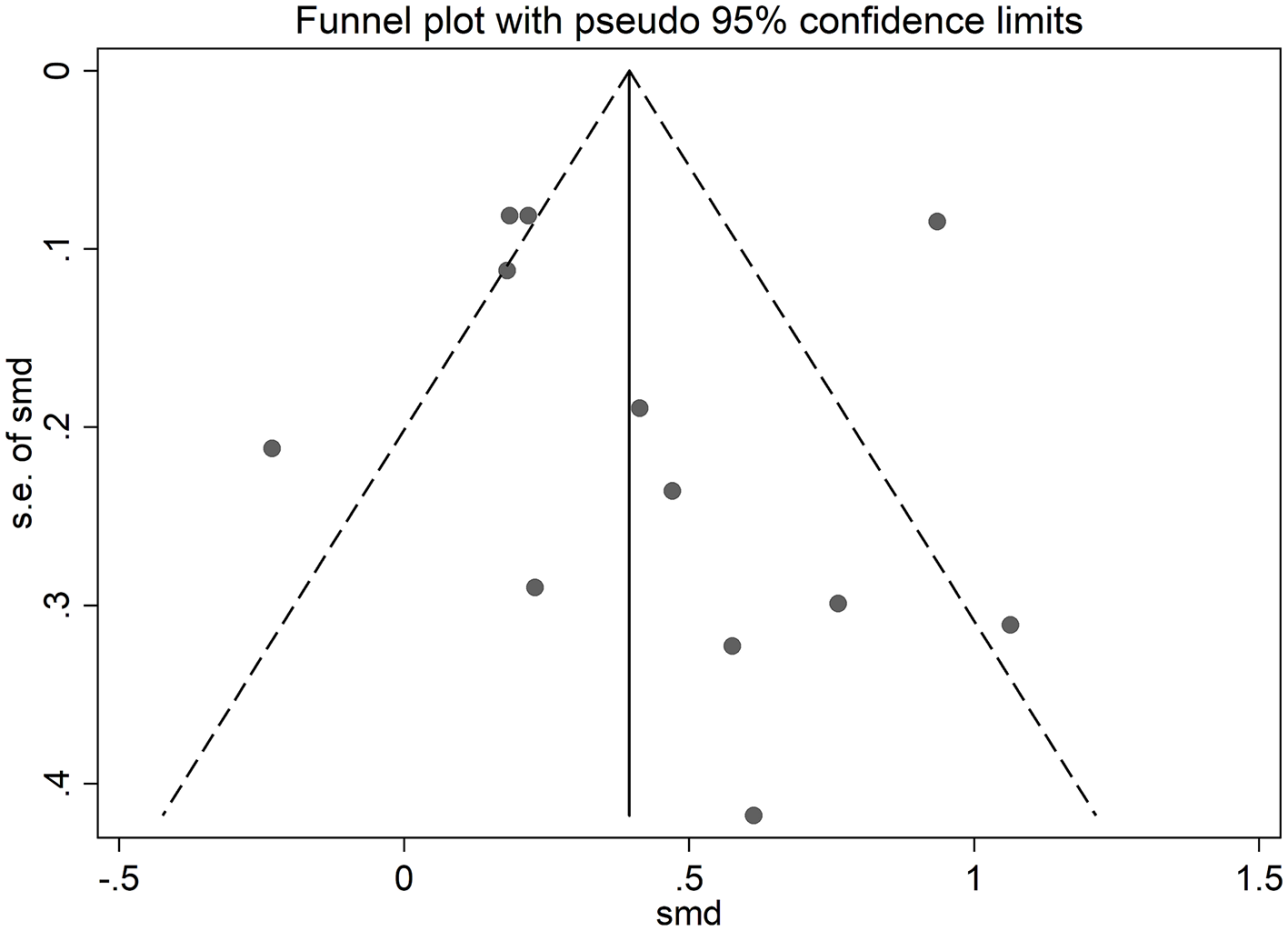
Funnel plot of standard mean difference against standard error for mean velocity. SE, Standard error of the mean difference; SMD, Standard mean difference.

**Figure 4:**
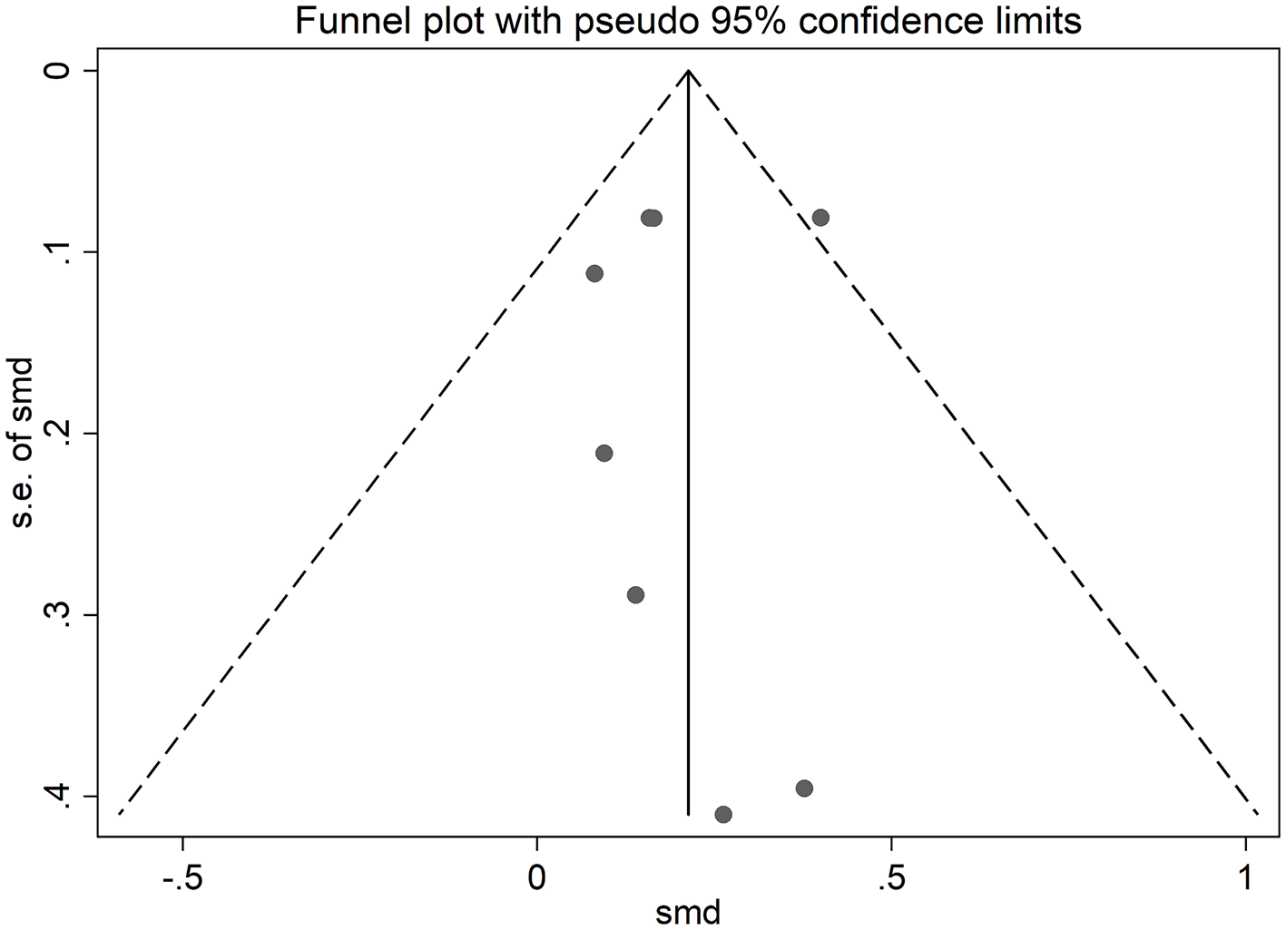
Funnel plot of standard mean difference against standard error for mean power output. SE, Standard error of the mean difference; SMD, Standard mean difference.

The risk of bias was assessed for the 12 included placebo-controlled crossover studies, all of which were rated as some concerns (Figure 2). The funnel plot showed slight asymmetry, suggesting potential publication bias (Figures 3, 4). Given the subjectivity of funnel plots, Egger’s linear regression was performed for MV and MPO during resistance exercises, which revealed no difference from zero (all *p* > 0.05), indicating no publication bias.

### 3.3 Meta-analysis results

#### 3.3.1 Mean velocity

**Figure 5:**
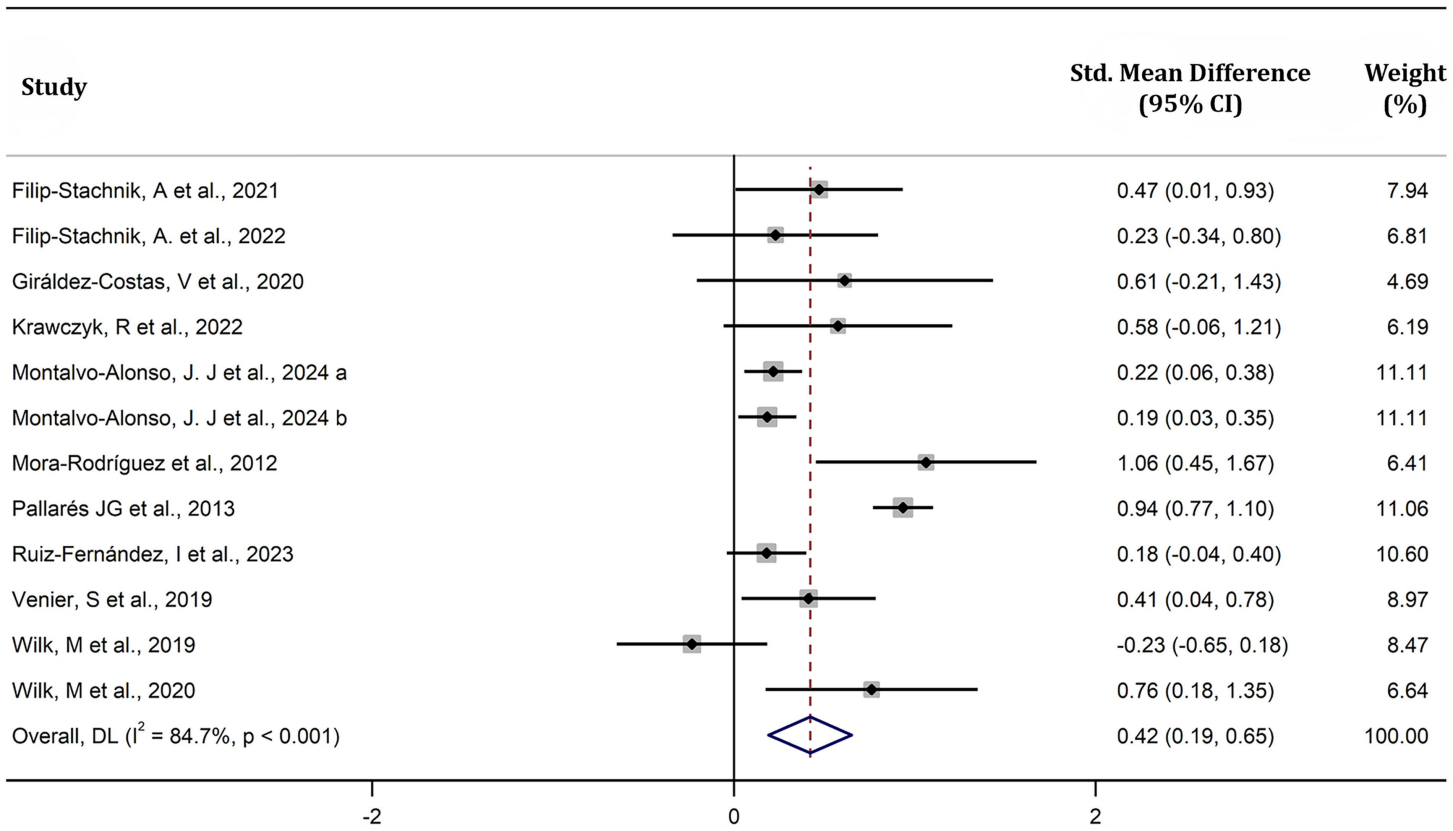
Effect of caffeine supplementation on mean velocity. The x-axis shows standardized mean differences (Hedge’s g) between caffeine and placebo conditions, with horizontal lines representing 95% confidence intervals (CI). “a” and “b” represent males and females, respectively.

**Figure 6:**
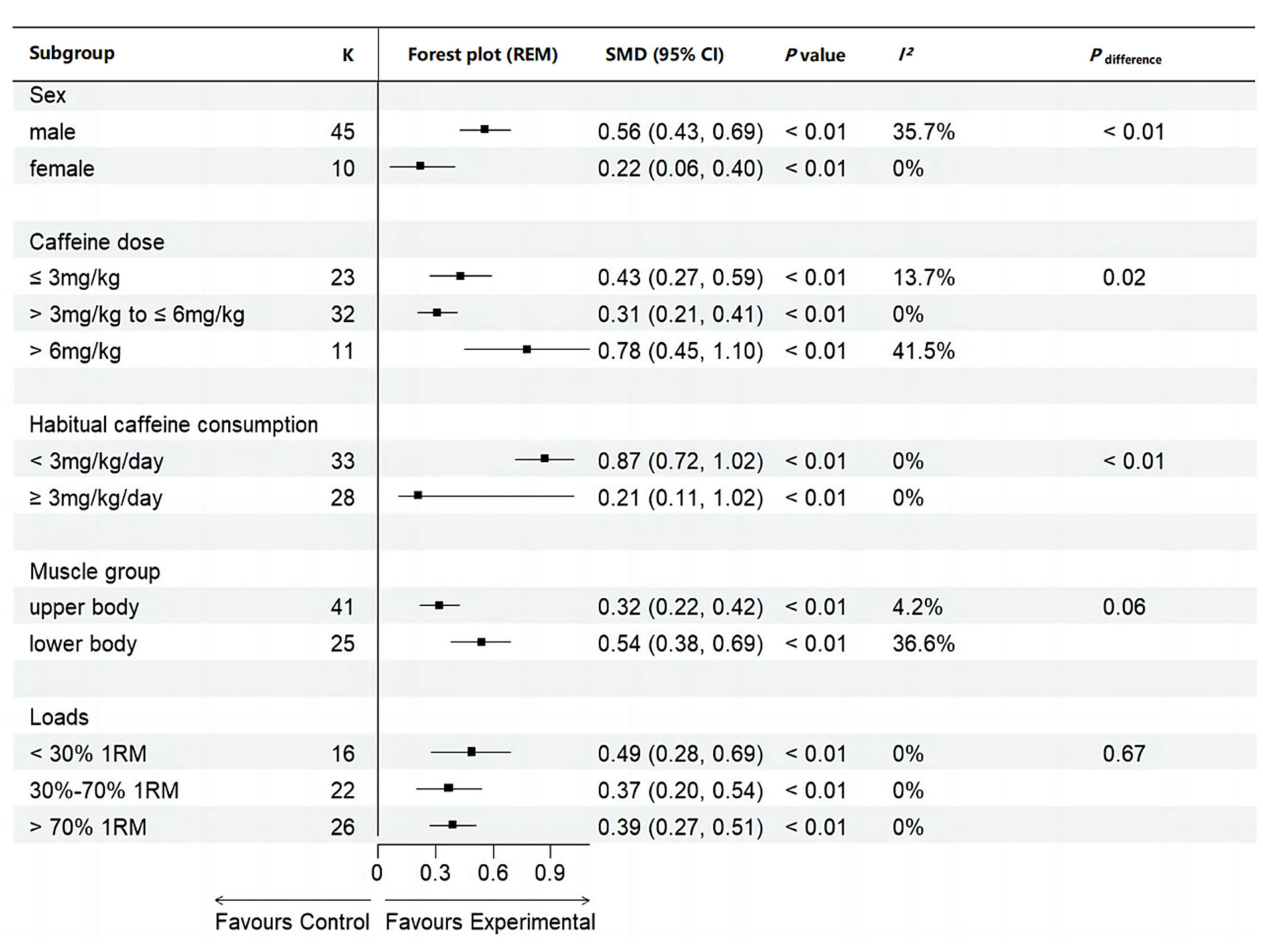
Subgroup analyses of mean velocity. K, the total number of effects included in the pooled effect size; SMD, Standardized Mean Difference; CI, confidence interval; *p* value, statistically significant *p* values for pooled results; *p*_difference_, *p* value of the difference between subgroups.

This meta-analysis demonstrated that caffeine significantly enhances MV during resistance exercises (SMD = 0.42, 95% CI = 0.19–0.65, *p* < 0.05, *I^2^* = 85%) (Figure 5). Subgroup analysis identified sex and habitual caffeine consumption as significant moderators (all subgroup differences *p* < 0.01) (Figure 6). Specifically, the effect size of caffeine on MV was larger in males (SMD = 0.56, 95% CI = 0.43–0.69, *p* < 0.01, *I^2^* = 36%) than females (SMD = 0.22, 95% CI = 0.06–0.40, *p* < 0.01, *I^2^* = 0%) (Figure 6). Naïve-to-mild caffeine consumers (< 3 mg/kg/day) showed greater improvements (SMD = 0.87, 95% CI = 0.72–1.02, *p* < 0.01, *I^2^* = 0%) compared to moderate-to-high caffeine consumers (≥ 3 mg/kg) (SMD = 0.21, 95% CI = 0.11–1.02, *p* < 0.01, *I^2^* = 0%) (Figure 6). Although caffeine significantly increased MV across all doses (high (> 6 mg/kg): SMD = 0.78, 95% CI = 0.45–1.10, *p* < 0.01, *I^2^* = 42%); moderate (> 3 mg/kg to ≤ 6 mg/kg) (SMD = 0.31, 95% CI = 0.21–0.41, *p* < 0.01, *I^2^* = 0%); low (≤ 3 mg/kg) (SMD = 0.43, 95% CI = 0.27–0.59, *p* < 0.01, *I^2^* = 14%) and (Figure 6); loads (low [< 30%1RM]: SMD = 0.49, 95% CI = 0.28–0.69, *p* < 0.01, *I^2^* = 0%; moderate [30–70%1RM]: SMD = 0.37, 95% CI = 0.20–0.54, *p* < 0.01, *I^2^* = 0%; high [> 70%1RM]: SMD = 0.39, 95% CI = 0.27–0.51, *p* < 0.01, *I^2^* = 0%) and muscle groups (upper body: SMD = 0.32, 95% CI = 0.22–0.42, *p* < 0.01, *I^2^* = 4%; lower body: SMD = 0.54, 95% CI = 0.38–0.69, *p* < 0.01, *I^2^* = 37%), no significant differences were observed between these subgroups (all *p* > 0.01) (Figure 6).

#### 3.3.2 Mean power output

**Figure 7:**
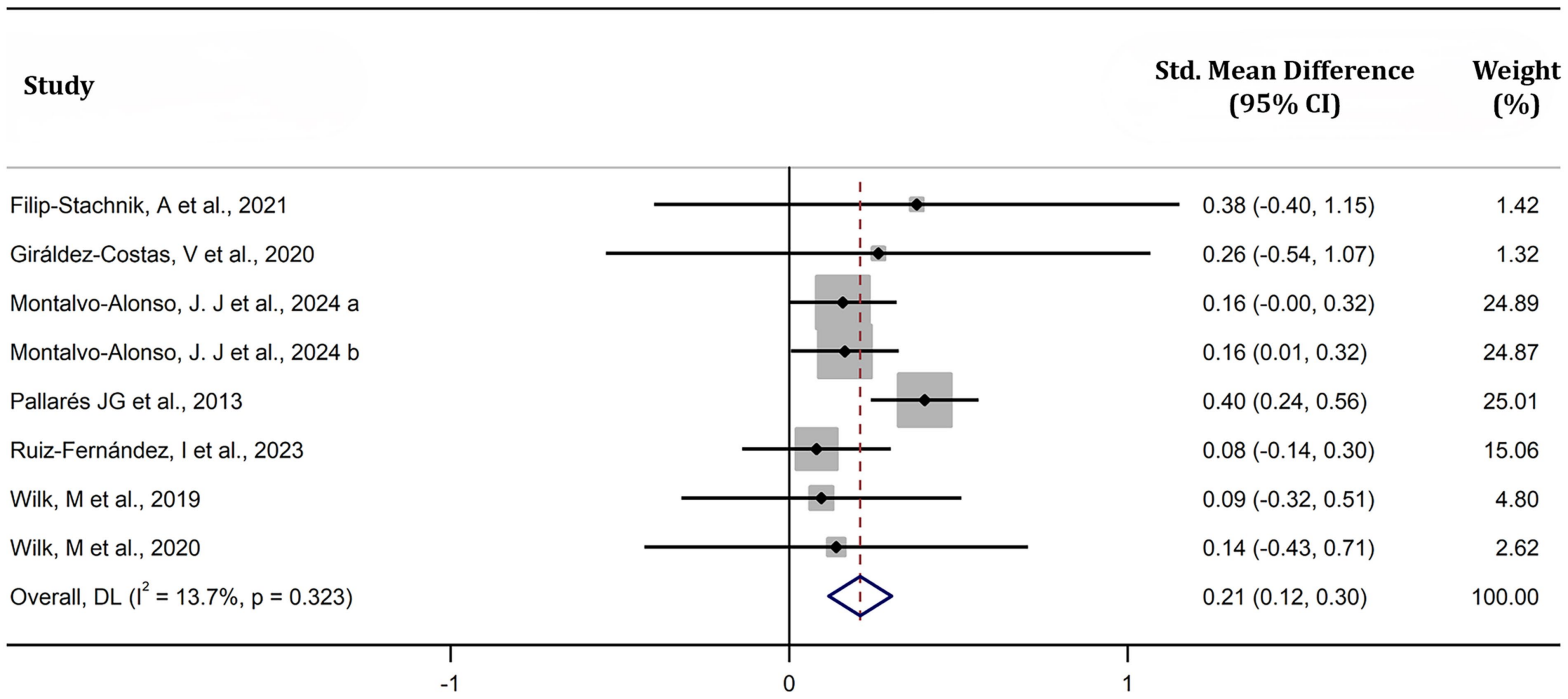
Effect of caffeine supplementation on mean power output. The x-axis shows standardized mean differences (Hedge’s g) between caffeine and placebo conditions, with horizontal lines representing 95% confidence intervals (CI). “a” and “b” represent males and females, respectively.

**Figure 8:**
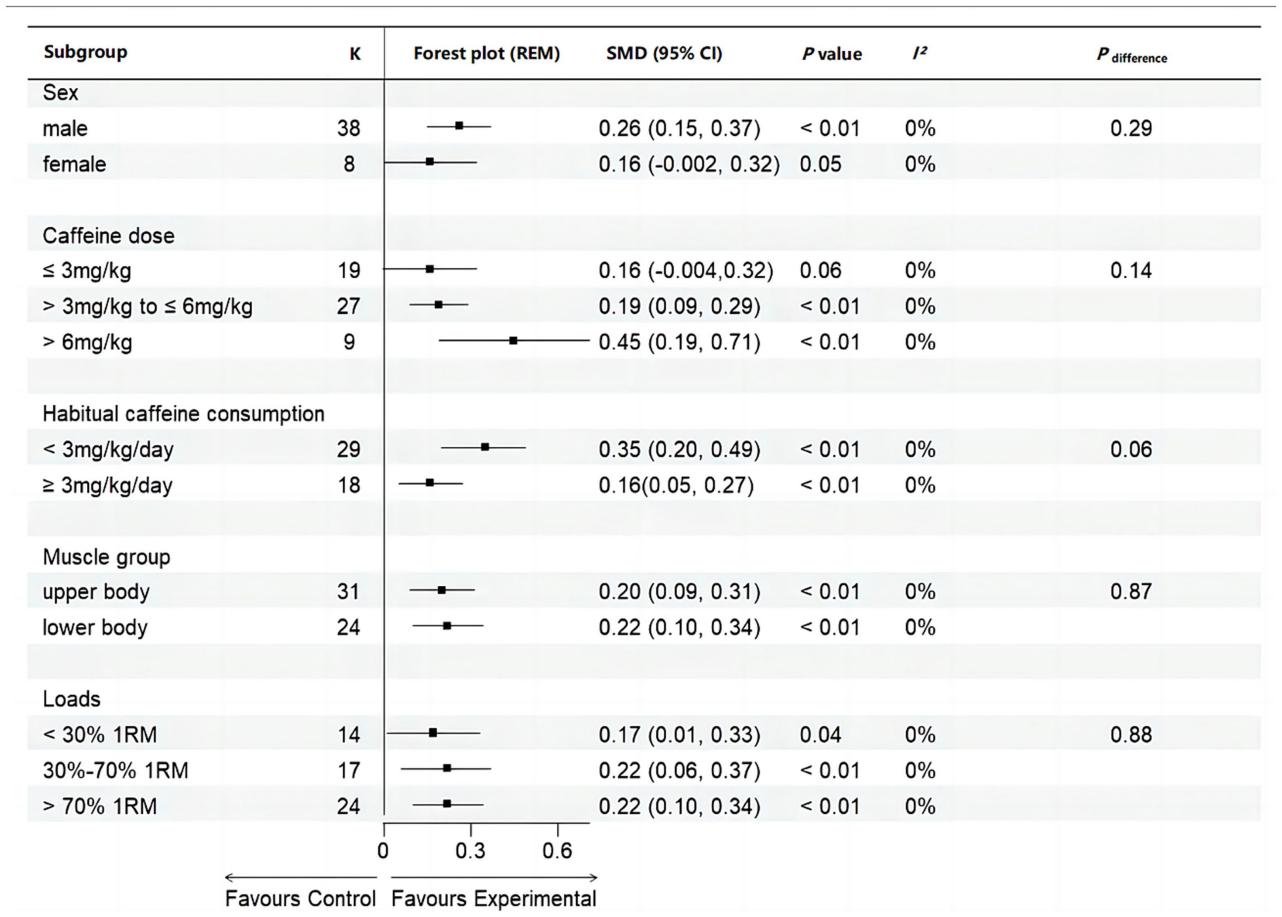
Subgroup analyses of mean power output. K, the total number of effects included in the pooled effect size; SMD, Standardized Mean Difference; CI, confidence interval; *p* value, statistically significant *p* values for pooled results; *p*_difference_: *p* value of the difference between subgroups.

This meta-analysis reported that caffeine significantly increased MPO during resistance exercises (SMD = 0.21, 95% CI: 0.12–0.30, *p* < 0.05, *I^2^* = 14%) (Figure 7). Subgroup analysis showed significant improvements across sexes (male: SMD = 0.26, 95% CI: 0.15–0.37, *p* < 0.01, *I^2^* = 0%; female: SMD = 0.16, 95% CI: −0.002–0.32, *p* = 0.05, *I^2^* = 0%), caffeine doses (low [≤ 3 mg/kg]: SMD = 0.16, 95% CI: −0.0004–0.32, *p* = 0.06, *I^2^* = 0%, moderate [> 3 mg/kg to ≤ 6 mg/kg]: SMD = 0.19, 95% CI: 0.09–0.31, *p* < 0.01, *I^2^* = 0%, high [> 6 mg/kg]: SMD = 0.45, 95% CI: 0.19–0.71, *p* < 0.01, *I^2^* = 0%), habitual caffeine consumption (naïve-to-mild [< 3 mg/kg]: SMD = 0.35, 95% CI = 0.20–0.49, *p* < 0.01, *I^2^* = 0%, moderate-to-high (≥ 3 mg/kg/day): SMD = 0.16, 95% CI = 0.05–0.27, *p* < 0.01, *I^2^* = 0%), muscle groups (upper body: SMD = 0.20, 95% CI: 0.09–0.31, *p* < 0.01, *I^2^* = 0%; lower body: SMD = 0.22, 95% CI: 0.10–0.34, *p* < 0.01, *I^2^* = 0%), and loads (low [< 30%1RM]: SMD = 0.17, 95% CI: 0.01–0.33, *p* = 0.04, *I^2^* = 0%, moderate [30–70%1RM]: SMD = 0.22, 95% CI: 0.06–0.37, *p* < 0.01, *I^2^* = 0%, high [> 70%1RM]: SMD = 0.22, 95% CI: 0.10–0.34, *p* < 0.01, *I^2^* = 0%) (Figure 8). However, no significant differences were observed between subgroups across these factors (all *p* > 0.05) (Figure 8).

### 3.4 Sensitivity analysis

Sensitivity analysis was conducted by sequentially excluding each included study, revealing that the effect of caffeine on MV and MPO remained significant (all *p* < 0.05) (Supplementary Figures S1–S20).

## 4 Discussion

The main findings showed that caffeine significantly improves both MV and MPO during resistance exercise for various sex (male and female), doses (≤3 mg/kg, > 3 mg/kg to ≤ 6 mg/kg, and > 6 mg/kg), habitual caffeine consumption (<3 mg/kg/day and ≥ 3 mg/kg/day), muscle groups (upper and lower bodies) and loads (low [<30% 1RM], moderate [30–70% 1RM], and high [>70% 1RM]) (all *p* < 0.05). Notably, the performance-enhancing effect of caffeine on MV was superior in males and habitual caffeine consumption < 3 mg/kg/day (all *p* < 0.05 for subgroup comparisons). These results further demonstrate the effectiveness of caffeine in enhancing muscular power during resistance exercises.

### 4.1 Muscular power outcomes

Our meta-analysis revealed that caffeine significantly enhances both MV and MPO during resistance exercises, with *small* effect sizes of 0.42 and 0.21, respectively (Figures 5, 6). The observations align with previous meta-analyses reporting comparable improvements (SMD = 0.62 for muscular power) (12). The ergogenic effects of caffeine may be attributed to several mechanisms: (a) increased excitability of the corticospinal tract (39), enhancing motor unit recruitment, firing frequency, and muscle fiber conduction velocity (40, 41); (b) antagonism of adenosine receptors, resulting in lower ratings of perceived exertion and pain perception (6, 13); and (c) enhanced function of sodium-potassium and calcium pumps, improving excitation-contraction coupling (42, 43). These mechanisms collectively enhance muscle force production and increase muscular power during resistance exercises. Our observed effect size for MV (SMD = 0.40; Figure 5) was lower than some prior reports (SMD = 0.80), possibly because we included studies with moderate-to-high habitual caffeine consumers (≥ 3 mg/kg/day) (20, 22, 23, 25), who may exhibit reduced responsiveness. Supporting this, our subgroup analysis revealed larger effects in naïve-to-mild caffeine consumers (SMD = 0.80) compared to moderate-to-high consumers (SMD = 0.19) (Figure 6). Recent reviews also indicate caffeine has a more pronounced effect on muscular power than on 1RM (13, 14). Our findings support this, with effect sizes for muscular power outcomes (SMD: 0.21–0.40) slightly exceeding those previously reported for 1RM (SMD: 0.17–0.20) (10, 11). This may be due to the lower external loads used in power-based testing, which may allow for greater neural and contractile improvements from caffeine intake (44). Overall, our findings reinforce that caffeine effectively improves muscular power during resistance exercise, particularly in individuals with low habitual caffeine consumption.

### 4.2 Potential factors

#### 4.2.1 Sex

Caffeine had a significantly greater effect on MV in males than females (SMD: 0.56 vs. 0.22) (Figure 6). Caffeine had a significantly greater effect on MV in males than females (SMD: 0.56 vs. 0.22) (Figure 6). This observation is consistent with recent previous meta-analyses reporting that caffeine significantly increased 1RM during resistance exercise in males (*p* = 0.01–0.03) but not in females (*p* = 0.29–0.57) (11, 45). One possible explanation is that hormonal fluctuations across the menstrual cycle, which may impair caffeine metabolism and reduce its ergogenic effects in women (46). However, as only two studies in our review exclusively included female groups (17, 20), the robustness of this conclusion is limited. Further research is needed to clarify sex-specific responses to caffeine during resistance exercise.

#### 4.2.2 Dose

Our subgroup analysis revealed a dose-dependent effect of caffeine on MV, with high doses (> 6 mg/kg) producing relatively larger effect sizes than moderate-to-low doses (1–6 mg/kg) (SMD: 0.78 vs. 0.31–0.43) (Figure 6). This aligns with a previous study where both 3 mg/kg and 6 mg/kg failed to increase MV during bench press at 90% 1RM, but 9 mg/kg was effective (28). Notably, most high-dose studies in our review involved moderate habitual caffeine consumers (≥ 3 mg/kg/day) (22, 25), making it unclear whether comparable effects would be observed in caffeine-naïve individuals. Nonetheless, these high-dose studies also reported relatively high incidence of side effects (9–12 mg/kg) (22, 25, 28), particularly tachycardia and heart palpitations (17–83%), anxiety or nervousness (13–83%), and increased urine output (25–62%). Another review likewise reported higher incidences of tachycardia and heart palpitations (83% vs. 12–24%) and headaches (30% vs. 5–14%) compared to lower doses (≤ 6 mg/kg) (47). Overall, although our results indicate that the side effects of high caffeine doses may not outweigh their ergogenic effects, future research should quantify the balance of benefits and risks across different populations. In practice, individual caffeine sensitivity must be considered when devising effective and safe personalized caffeine intake strategies.

#### 4.2.3 Habitual caffeine consumption

Subgroup analysis reported that caffeine improves MV and MPO in both groups, but the effect (MV: 0.87 vs. 0.21, MPO: 0.35 vs. 0.16) is significantly larger in naïve-to-mild consumers (< 3 mg/kg/day) than in moderate-to-high consumers (≥ 3 mg/kg/day) (Figures 6, 8). This may be due to tolerance developed through habitual caffeine intake, potentially due to upregulation of adenosine receptors (15, 48, 49). Previous research has indicated that consuming of 3 mg/kg/day of caffeine for 15 days can increase individual tolerance, thereby reducing its ergogenic effect on peak cycling power during incremental exercise (29). Therefore, athletes are advised to limit daily caffeine intake to preserve its acute performance-enhancing effects during competition.

#### 4.2.4 Muscle group

Muscle group significantly moderated the effect of caffeine on MV during resistance exercise, with larger effect sizes observed for lower body compared to upper body (SMD: 0.54 vs. 0.32; Figure 6). This aligns with a previous meta-analysis showing larger caffeine-induced improvements in lower-body MVC strength (50). This difference may be attributed to variations in muscle mass. It has been reported that larger muscle groups, such as the knee extensors, have more capacity for improvement in voluntary activation levels (85–95%) compared to smaller groups like the elbow flexors (90–99%) (30). Through its antagonistic action in the central nervous system, caffeine enhances neural signaling (51), resulting in greater activation of lower body muscles compared to the upper body.

#### 4.2.5 Loads

A previous meta-analysis found that caffeine enhances MV across a wide range of loads (25–90% 1RM) during resistance exercise (12). Consistent with this, our results revealed that caffeine significantly increased MV and MPO, regardless of load (Figures 7, 8). Considering that different loads target distinct training adaptations—low (0–30% 1RM) for power-focused movements such as bench press throws or vertical jumps, moderate (40–70% 1RM) for muscular power in exercises like bench press and back squat, and high (>70% 1RM) for maximum strength (35, 36)—our findings suggests that caffeine consumption is an effective strategy to boost performance across all resistance training intensities.

### 4.3 Limitations and future considerations

Several limitations of this study should be acknowledged. Firstly, we did not consider the influence of participants’ genetic types on caffeine responsiveness. Existing research has found that carriers of the AA genotype may be more sensitive to caffeine’s ergogenic effects compared to those with CC or AC genotypes (15, 52). Secondly, due to the limited number of available studies, we were unable to conduct subgroup analyses based on different forms of caffeine intake (e.g., capsules, tablets, and gum). Notably, caffeine gum may offer advantages such as a faster absorption rate and a lower incidence of side effects (27, 37, 53). Our previous study also found that caffeinated chewing gum (3 mg/kg) significantly improved maximal strength during resistance exercises (27), with a lower incidence of side effects (e.g., muscle soreness: 0% vs. 24%; insomnia: 6.3% vs. 34%) compared to a systematic review on low dose caffeine (0–3 mg/kg) (47). Future research should directly compare the effects of different intake forms to determine their effects on muscular power outcomes. Thirdly, since the studies included in our review primarily recruited young, healthy, resistance-trained men, these findings may not be applicable to other populations, such as sedentary individuals, injured athletes, women, adolescents, or older adults. Further research is needed to address these gaps. Fourthly, since none of the included studies provided participant’s detailed dietary records during the experimental sessions, the possibility of additional caffeine sources (e.g., energy drinks and chocolate), cannot be excluded and may have influenced our results. Finally, although high-dose caffeine was associated with larger effect sizes, our analysis did not assess the incidence of side effects. This limits the practical significance of the results. Future research should examine the balance between performance benefits and adverse effects to better guide dosing recommendations.

## 5 Conclusion

This meta-analysis demonstrated that caffeine significantly enhances MV and MPO during resistance exercises across all loads. Subgroup analysis indicated that sex and habitual caffeine consumption, both modulate the effectiveness of caffeine on muscular power outcomes. Greater ergogenic effects were observed among males, and in individuals with habitual caffeine consumption < 3 mg/kg/day. These findings underscore the importance of tailoring caffeine supplementation strategies to maximize muscular power gains during resistance exercise.

## Data Availability

The original contributions presented in the study are included in the article/Supplementary material, further inquiries can be directed to the corresponding author.

## References

[1] Redman KJ Wade L Whitley R Connick MJ Kelly VG Beckman EM. Seasonal muscular power changes: considerations of concurrent resistance and field-based training in professional Rugby league. J Strength Cond Res. (2024) 38:1620–6. doi: 10.1519/JSC.0000000000004842, PMID: 39074167

[2] Douglas J Ross A Martin JC. Maximal muscular power: lessons from sprint cycling. Sports Med Open. (2021) 7:48. doi: 10.1186/s40798-021-00341-7, PMID: 34268627 PMC8282832

[3] Belcher DJ Sousa CA Carzoli JP Johnson TK Helms ER Visavadiya NP . Time course of recovery is similar for the back squat, bench press, and deadlift in well-trained males. Appl Physiol Nutr Metab. (2019) 44:1033–42. doi: 10.1139/apnm-2019-0004, PMID: 30779596

[4] Cowley N Nicholson V Timmins R Munteanu G Wood T García-Ramos A . The effects of percentage-based, rating of perceived exertion, repetitions in reserve, and velocity-based training on performance and fatigue responses. J Strength Cond Res. (2025) 39:e516–29. doi: 10.1519/JSC.0000000000005026, PMID: 39787033

[5] Wilk M Krzysztofik M Filip A Zajac A Bogdanis GC Lockie RG. Short-term blood flow restriction increases power output and Bar velocity during the bench press. J Strength Cond Res. (2022) 36:2082–8. doi: 10.1519/JSC.0000000000003649, PMID: 32379236

[6] Grgic J Sabol F Venier S Mikulic I Bratkovic N Schoenfeld BJ . What dose of caffeine to use: acute effects of 3 doses of caffeine on muscle endurance and strength. Int J Sports Physiol Perform. (2020) 15:470–7. doi: 10.1123/ijspp.2019-0433, PMID: 31575825

[7] Haugen ME Vårvik FT Grgic J Studsrud H Austheim E Zimmermann EM . Effect of isolated and combined ingestion of caffeine and citrulline malate on resistance exercise and jumping performance: a randomized double-blind placebo-controlled crossover study. Eur J Nutr. (2023) 62:2963–75. doi: 10.1007/s00394-023-03212-x, PMID: 37450275 PMC10468939

[8] Weinstein Y Ovadia Y Weinstein B Weinstein A. The effects of amorphous calcium carbonate (ACC) supplementation on resistance exercise performance in women. Nutrients. (2023) 15:538. doi: 10.3390/nu15030538, PMID: 36771244 PMC9919417

[9] Cappelletti S Piacentino D Sani G Aromatario M. Caffeine: cognitive and physical performance enhancer or psychoactive drug? Curr Neuropharmacol. (2015) 13:71–88. doi: 10.2174/1570159X13666141210215655, PMID: 26074744 PMC4462044

[10] Bilondi HT Valipour H Khoshro S Jamilian P Ostadrahimi A Zarezadeh M. The effect of caffeine supplementation on muscular strength and endurance: a meta-analysis of meta-analyses. Heliyon. (2024) 10:e35025. doi: 10.1016/j.heliyon.2024.e35025, PMID: 39170391 PMC11336343

[11] Grgic J Trexler ET Lazinica B Pedisic Z. Effects of caffeine intake on muscle strength and power: a systematic review and meta-analysis. J Int Soc Sports Nutr. (2018) 15:11. doi: 10.1186/s12970-018-0216-0, PMID: 29527137 PMC5839013

[12] Raya-González J Rendo-Urteaga T Domínguez R Castillo D Rodríguez-Fernández A Grgic J. Acute effects of caffeine supplementation on movement velocity in resistance exercise: a systematic review and Meta-analysis. Sports Med. (2020) 50:717–29. doi: 10.1007/s40279-019-01211-9, PMID: 31643020

[13] Grgic J Mikulic P Schoenfeld BJ Bishop DJ Pedisic Z. The influence of caffeine supplementation on resistance exercise: a review. Sports Med. (2019) 49:17–30. doi: 10.1007/s40279-018-0997-y, PMID: 30298476

[14] Grgic J. Effects of caffeine on resistance exercise: a review of recent research. Sports Med. (2021) 51:2281–98. doi: 10.1007/s40279-021-01521-x, PMID: 34291426

[15] Chen B Ding L Qin Q Lei TH Girard O Cao Y. Effect of caffeine ingestion on time trial performance in cyclists: a systematic review and meta-analysis. J Int Soc Sports Nutr. (2024) 21:2363789. doi: 10.1080/15502783.2024.2363789, PMID: 38836626 PMC11155427

[16] Guest NS VanDusseldorp TA Nelson MT Grgic J Schoenfeld BJ Jenkins NDM . International society of sports nutrition position stand: caffeine and exercise performance. J Int Soc Sports Nutr. (2021) 18:1. doi: 10.1186/s12970-020-00383-4, PMID: 33388079 PMC7777221

[17] Montalvo-Alonso JJ Ferragut C Del Val-Manzano M Valadés D Roberts J Pérez-López A. Sex differences in the ergogenic response of acute caffeine intake on muscular strength, power and endurance performance in resistance-trained individuals: a randomized controlled trial. Nutrients. (2024) 16:760. doi: 10.3390/nu16111760, PMID: 38892692 PMC11174740

[18] Ruiz-Fernández I Valadés D Dominguez R Ferragut C Pérez-López A. Load and muscle group size influence the ergogenic effect of acute caffeine intake in muscular strength, power and endurance. Eur J Nutr. (2023) 62:1783–94. doi: 10.1007/s00394-023-03109-9, PMID: 36840816

[19] Krawczyk R Krzysztofik M Kostrzewa M Komarek Z Wilk M Del Coso J . Preliminary research towards acute effects of different doses of caffeine on strength-power performance in highly trained judo athletes. Int J Environ Res Public Health. (2022) 19:868. doi: 10.3390/ijerph19052868, PMID: 35270556 PMC8910536

[20] Filip-Stachnik A Krzysztofik M Del Coso J Wilk M. Acute effects of two caffeine doses on bar velocity during the bench press exercise among women habituated to caffeine: a randomized, crossover, double-blind study involving control and placebo conditions. Eur J Nutr. (2022) 61:947–55. doi: 10.1007/s00394-021-02708-8, PMID: 34664106 PMC8854307

[21] Filip-Stachnik A Krzysztofik M Kaszuba M Leznicka K Kostrzewa M Del Coso J . Effects of acute caffeine intake on power output and movement velocity during a multiple-set bench press exercise among mild caffeine users. J Hum Kinet. (2021) 78:219–28. doi: 10.2478/hukin-2021-0044, PMID: 34025879 PMC8120957

[22] Filip-Stachnik A Krzysztofik M Del Coso J Wilk M. Acute effects of high doses of caffeine on Bar velocity during the bench press throw in athletes habituated to caffeine: a randomized, double-blind and crossover study. J Clin Med. (2021) 10:380. doi: 10.3390/jcm10194380, PMID: 34640398 PMC8509759

[23] Wilk M Filip A Krzysztofik M Gepfert M Zajac A Del Coso J. Acute caffeine intake enhances mean power output and Bar velocity during the bench press throw in athletes habituated to caffeine. Nutrients. (2020) 12:406. doi: 10.3390/nu12020406, PMID: 32033103 PMC7071256

[24] Giráldez-Costas V González-García J Lara B Coso JD Wilk M Salinero JJ. Caffeine increases muscle performance during a bench press training session. J Hum Kinet. (2020) 74:185–93. doi: 10.2478/hukin-2020-0024, PMID: 33312286 PMC7706635

[25] Wilk M Filip A Krzysztofik M Maszczyk A Zajac A. The acute effect of various doses of caffeine on power output and velocity during the bench press exercise among athletes habitually using caffeine. Nutrients. (2019) 11:1465. doi: 10.3390/nu11071465, PMID: 31252655 PMC6682895

[26] Ferreira TT Silva JVFD Bueno NB. Effects of caffeine supplementation on muscle endurance, maximum strength, and perceived exertion in adults submitted to strength training: a systematic review and meta-analyses. Crit Rev Food Sci Nutr. (2020) 61:25871–2600. doi: 10.1080/10408398.2020.1781051 32551869

[27] Ding L Liu J Yao Y Guo L Chen B Cao Y . Caffeinated chewing gum enhances maximal strength and muscular endurance during bench press and back squat exercises in resistance-trained men. Front Nutr. (2025) 12:552. doi: 10.3389/fnut.2025.1540552, PMID: 39944953 PMC11813785

[28] Pallarés JG Fernández-Elías VE Ortega JF Muñoz G Muñoz-Guerra J Mora-Rodríguez R. Neuromuscular responses to incremental caffeine doses: performance and side effects. Med Sci Sports Exerc. (2013) 45:2184–92. doi: 10.1249/MSS.0b013e31829a6672, PMID: 23669879

[29] Lara B Ruiz-Moreno C Salinero JJ Del Coso J Sandbakk Ø. Time course of tolerance to the performance benefits of caffeine. PLoS One. (2019) 14:e0210275. doi: 10.1371/journal.pone.0210275 30673725 PMC6343867

[30] Shield A Zhou S. Assessing voluntary muscle activation with the twitch interpolation technique. Sports Med. (2004) 34:253–67. doi: 10.2165/00007256-200434040-00005, PMID: 15049717

[31] Moher D Shamseer L Clarke M Ghersi D Liberati A Petticrew M . Preferred reporting items for systematic review and meta-analysis protocols (PRISMA-P) 2015 statement. Syst Rev. (2015) 4:1. doi: 10.1186/2046-4053-4-1, PMID: 25554246 PMC4320440

[32] Cohen J. Statistical power for the Behavioural sciences: Statistical power analysis for the behavioral sciences. New York: Routledge. (1988).

[33] Higgins JP Thompson SG. Quantifying heterogeneity in a meta-analysis. Stat Med. (2002) 21:1539–58. doi: 10.1002/sim.1186, PMID: 12111919

[34] Filip A Wilk M Krzysztofik M Del Coso J. Inconsistency in the ergogenic effect of caffeine in athletes who regularly consume caffeine: is it due to the disparity in the criteria that defines habitual caffeine intake? Nutrients. (2020) 12:1087. doi: 10.3390/nu12041087, PMID: 32326386 PMC7230656

[35] Swinton PA Schoenfeld BJ Murphy A. Dose-response modelling of resistance exercise across outcome domains in strength and conditioning: a Meta-analysis. Sports Med. (2024) 54:1579–94. doi: 10.1007/s40279-024-02006-3, PMID: 38652410 PMC11239729

[36] Soriano MA Suchomel TJ Marín PJ. The optimal load for maximal power production during upper-body resistance exercises: a Meta-analysis. Sports Med. (2017) 47:757–68. doi: 10.1007/s40279-016-0626-6, PMID: 27699699

[37] Venier S Grgic J Mikulic P. Acute enhancement of jump performance, muscle strength, and power in resistance-trained men after consumption of caffeinated chewing gum. Int J Sports Physiol Perform. (2019) 14:1415–21. doi: 10.1123/ijspp.2019-0098, PMID: 30958062

[38] Mora-Rodríguez R García Pallarés J López-Samanes Á Ortega JF Fernández-Elías VE. Caffeine ingestion reverses the circadian rhythm effects on neuromuscular performance in highly resistance-trained men. PLoS One. (2012) 7:e33807. doi: 10.1371/journal.pone.0033807, PMID: 22496767 PMC3319538

[39] Sawynok J. Adenosine receptor targets for pain. Neuroscience. (2016) 338:1–18. doi: 10.1016/j.neuroscience.2015.10.031, PMID: 26500181

[40] Snyder BJ Fry WR. Effect of verbal instruction on muscle activity during the bench press exercise. J Strength Cond Res. (2012) 26:2394–400. doi: 10.1519/JSC.0b013e31823f8d11, PMID: 22076100

[41] Edwards RH. Human muscle function and fatigue. Ciba found Symp. (1981) 82:1–18. 10.1002/9780470715420.ch1 6117420

[42] Kamimori GH Karyekar CS Otterstetter R Cox DS Balkin TJ Belenky GL . The rate of absorption and relative bioavailability of caffeine administered in chewing gum versus capsules to normal healthy volunteers. Int J Pharm. (2002) 234:159–67. doi: 10.1016/S0378-5173(01)00958-9, PMID: 11839447

[43] Davis JK Green JM. Caffeine and anaerobic performance: ergogenic value and mechanisms of action. Sports Med. (2009) 39:813–32. doi: 10.2165/11317770-000000000-00000, PMID: 19757860

[44] Ba A Pa T He B. Quadriceps EMG/force relationship in knee extension and leg press. Med Sci Sports Exerc. (2000) 32:459–63. doi: 10.1097/00005768-200002000-00030 10694132

[45] Wu W Chen Z Zhou H Wang L Li X Lv Y . Effects of acute ingestion of caffeine capsules on muscle strength and muscle endurance: a systematic review and meta-analysis. Nutrients. (2024) 16:1146. doi: 10.3390/nu16081146, PMID: 38674836 PMC11054210

[46] Ouergui I Delleli S Bridge CA Messaoudi H Chtourou H Ballmann CG . Acute effects of caffeine supplementation on taekwondo performance: the influence of competition level and sex. Sci Rep. (2023) 13:13795. doi: 10.1038/s41598-023-40365-5, PMID: 37612360 PMC10447555

[47] de Souza JG Del Coso J Fonseca FS Silva BVC de Souza DB da Silva Gianoni RL . Risk or benefit? Side effects of caffeine supplementation in sport: a systematic review. Eur J Nutr. (2022) 61:3823–34. doi: 10.1007/s00394-022-02874-3, PMID: 35380245

[48] Fredholm BB. Adenosine actions and adenosine receptors after 1 week treatment with caffeine. Acta Physiol Scand. (1982) 115:283–6. doi: 10.1111/j.1748-1716.1982.tb07078.x, PMID: 6291335

[49] Nikodijević O Jacobson KA Daly JW. Locomotor activity in mice during chronic treatment with caffeine and withdrawal. Pharmacol Biochem Behav. (1993) 44:199–216. doi: 10.1016/0091-3057(93)90299-9, PMID: 7679219 PMC3557839

[50] Warren GL Park ND Maresca RD McKibans KI Millard-Stafford ML. Effect of caffeine ingestion on muscular strength and endurance: a meta-analysis. Med Sci Sports Exerc. (2010) 42:1375–87. doi: 10.1249/MSS.0b013e3181cabbd8, PMID: 20019636

[51] Limasilva AE Cristinasouza G Silvacavalcante MD Bertuzzi R Bishop DJ. Caffeine during high-intensity whole-body exercise: an integrative approach beyond the central nervous system. Nutrients. (2021) 13:2503. doi: 10.3390/nu13082503 34444663 PMC8400708

[52] Perera V Gross AS McLachlan AJ. Influence of environmental and genetic factors on CYP1A2 activity in individuals of south Asian and European ancestry. Clin Pharmacol Ther. (2012) 92:511–9. doi: 10.1038/clpt.2012.139, PMID: 22948892

[53] Barreto G Loureiro LMR Reis CEG Saunders B. Effects of caffeine chewing gum supplementation on exercise performance: a systematic review and meta-analysis. Eur J Sport Sci. (2023) 23:714–25. doi: 10.1080/17461391.2022.2049885, PMID: 35239468

