# Supplementary figures and images for: Effects of acute caffeine intake on muscular power during resistance exercise: a systematic review and meta-analysis
Source: Front Nutr. 2025 Oct 7;12:1686283. doi: 10.3389/fnut.2025.1686283 (PMC12537405; doi:10.3389/fnut.2025.1686283)

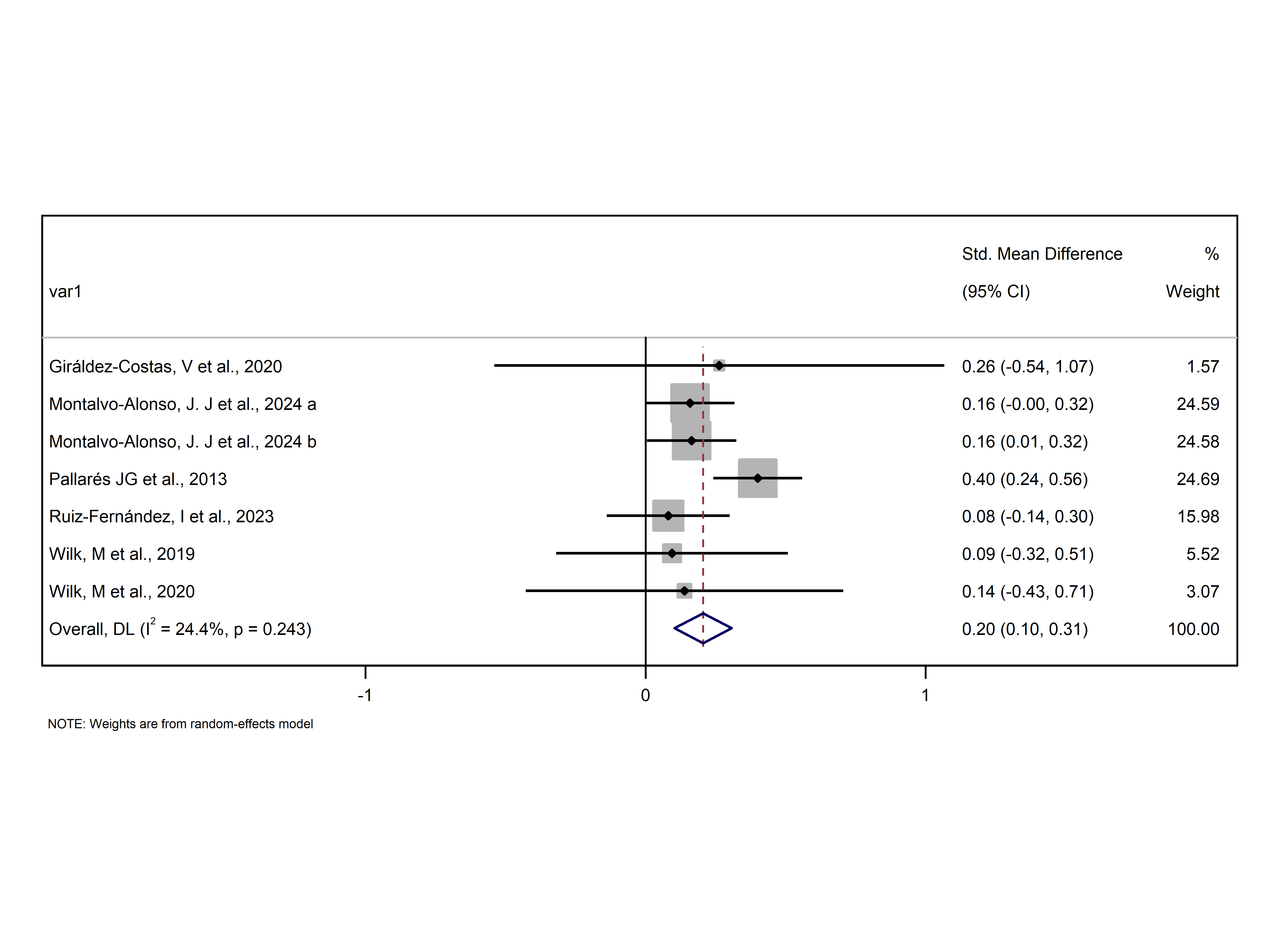

Supplement: Supplementary file 1 [file Data_Sheet_1.ZIP › S1.tif]

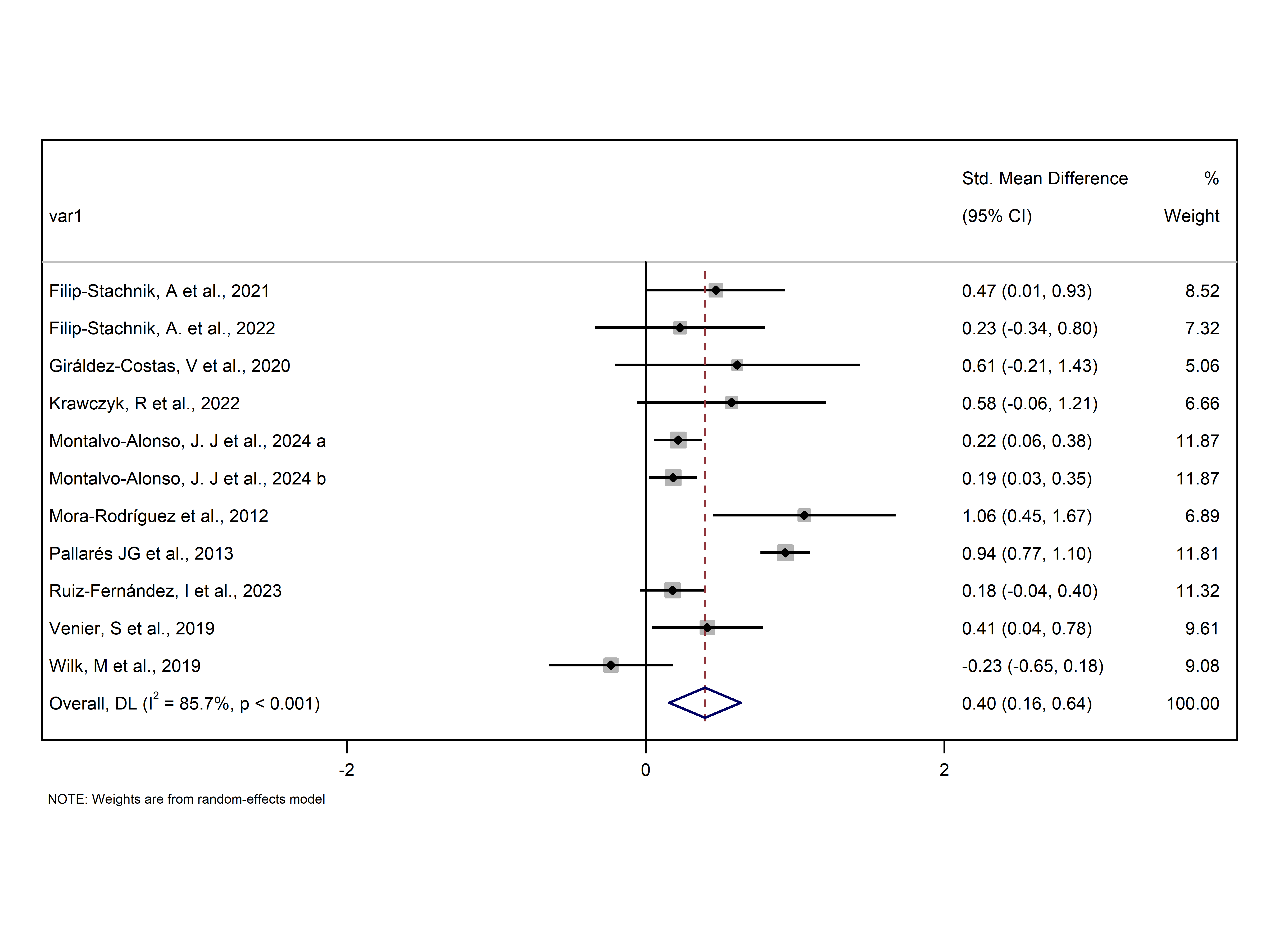

Supplement: Supplementary file 1 [file Data_Sheet_1.ZIP › S10.tif]

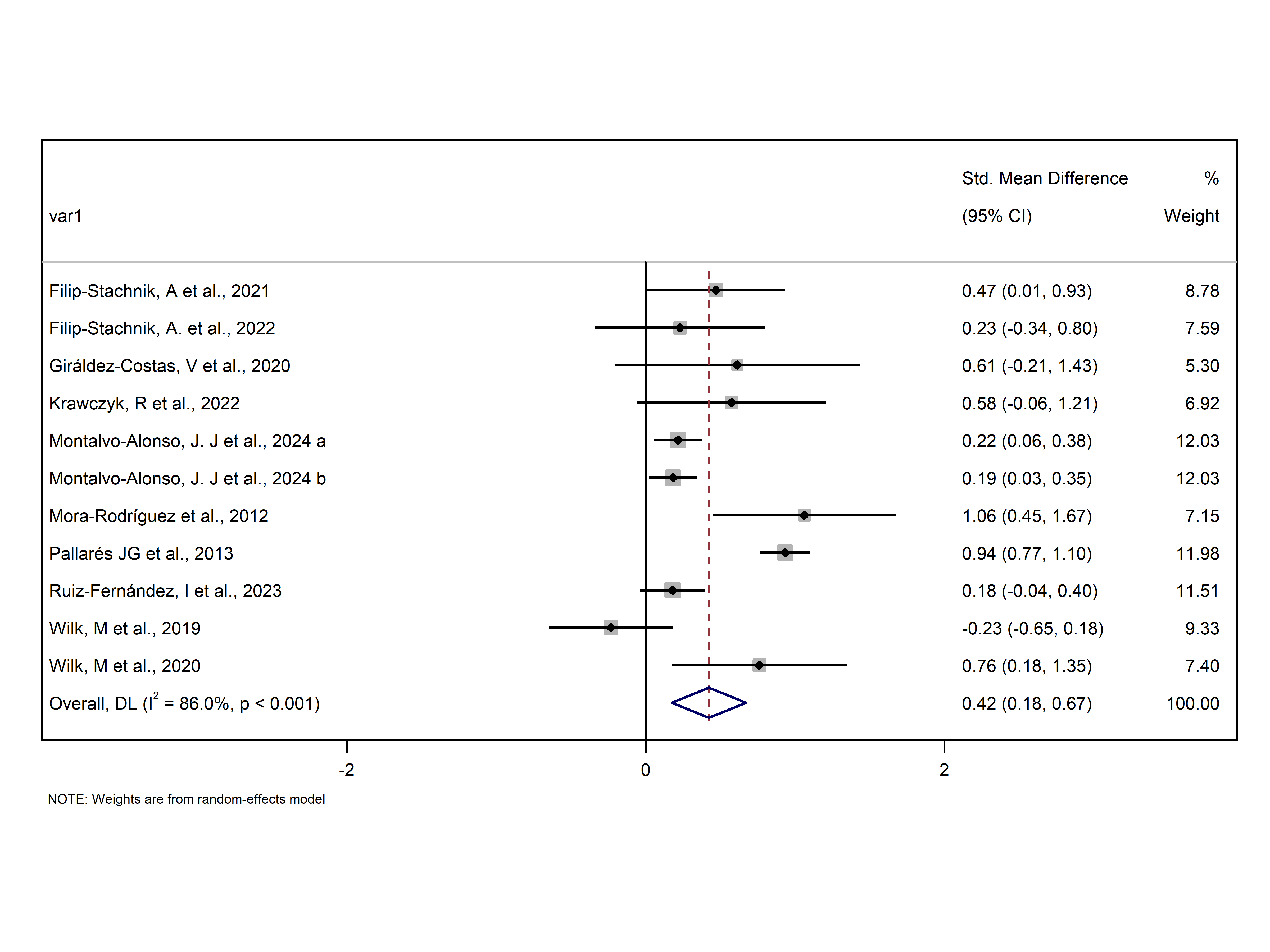

Supplement: Supplementary file 1 [file Data_Sheet_1.ZIP › S11.tif]

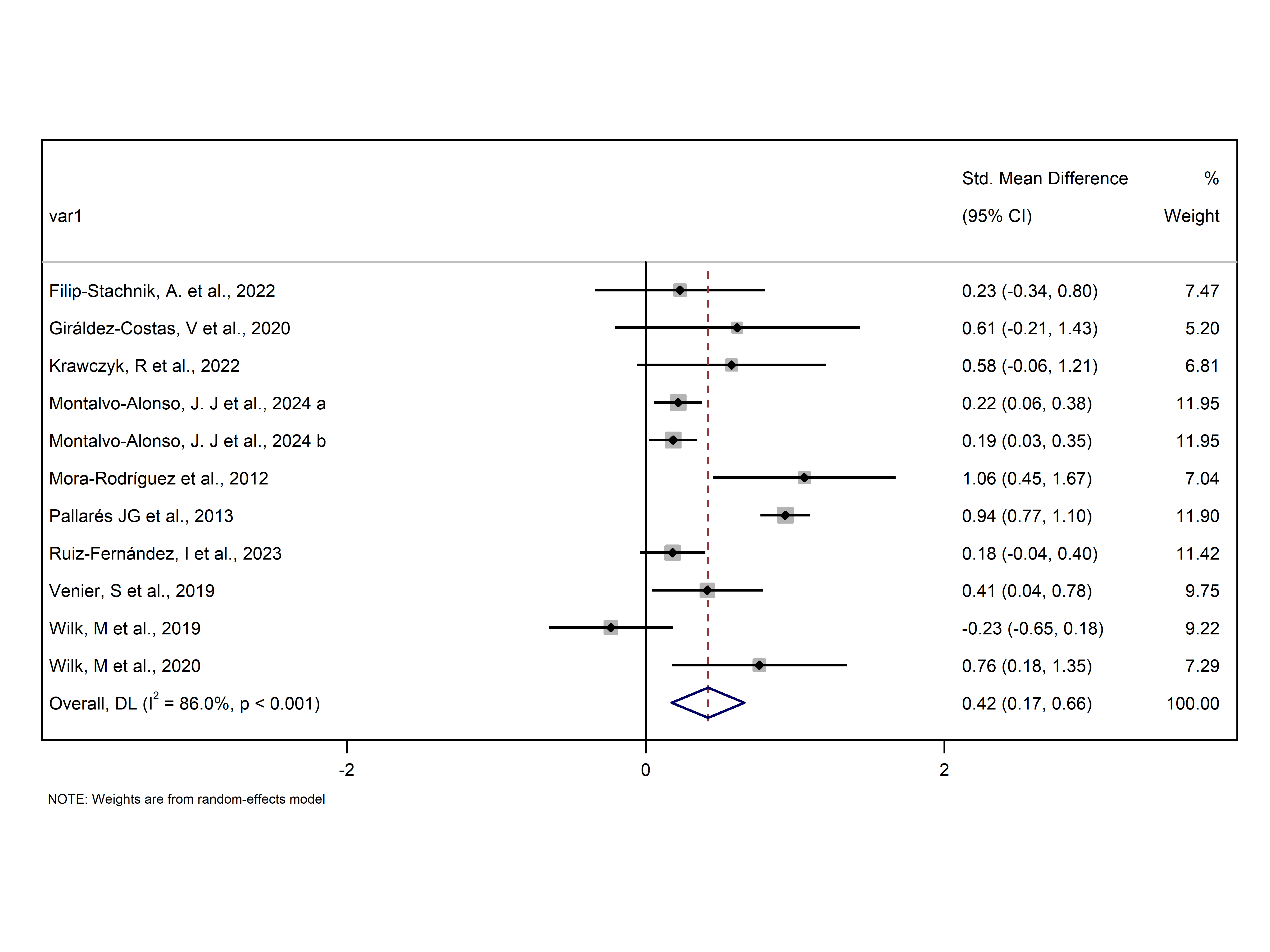

Supplement: Supplementary file 1 [file Data_Sheet_1.ZIP › S12.tif]

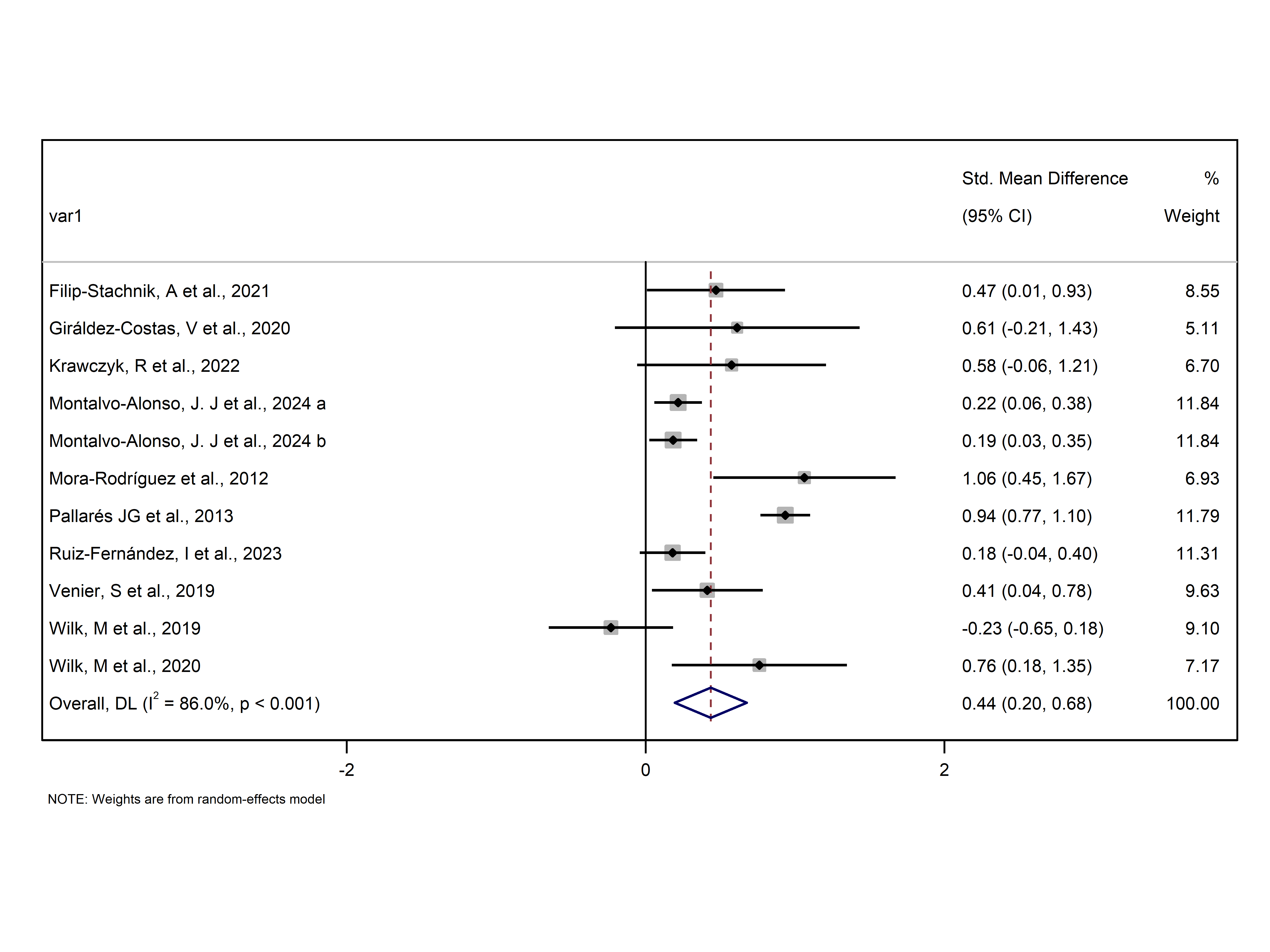

Supplement: Supplementary file 1 [file Data_Sheet_1.ZIP › S13.tif]

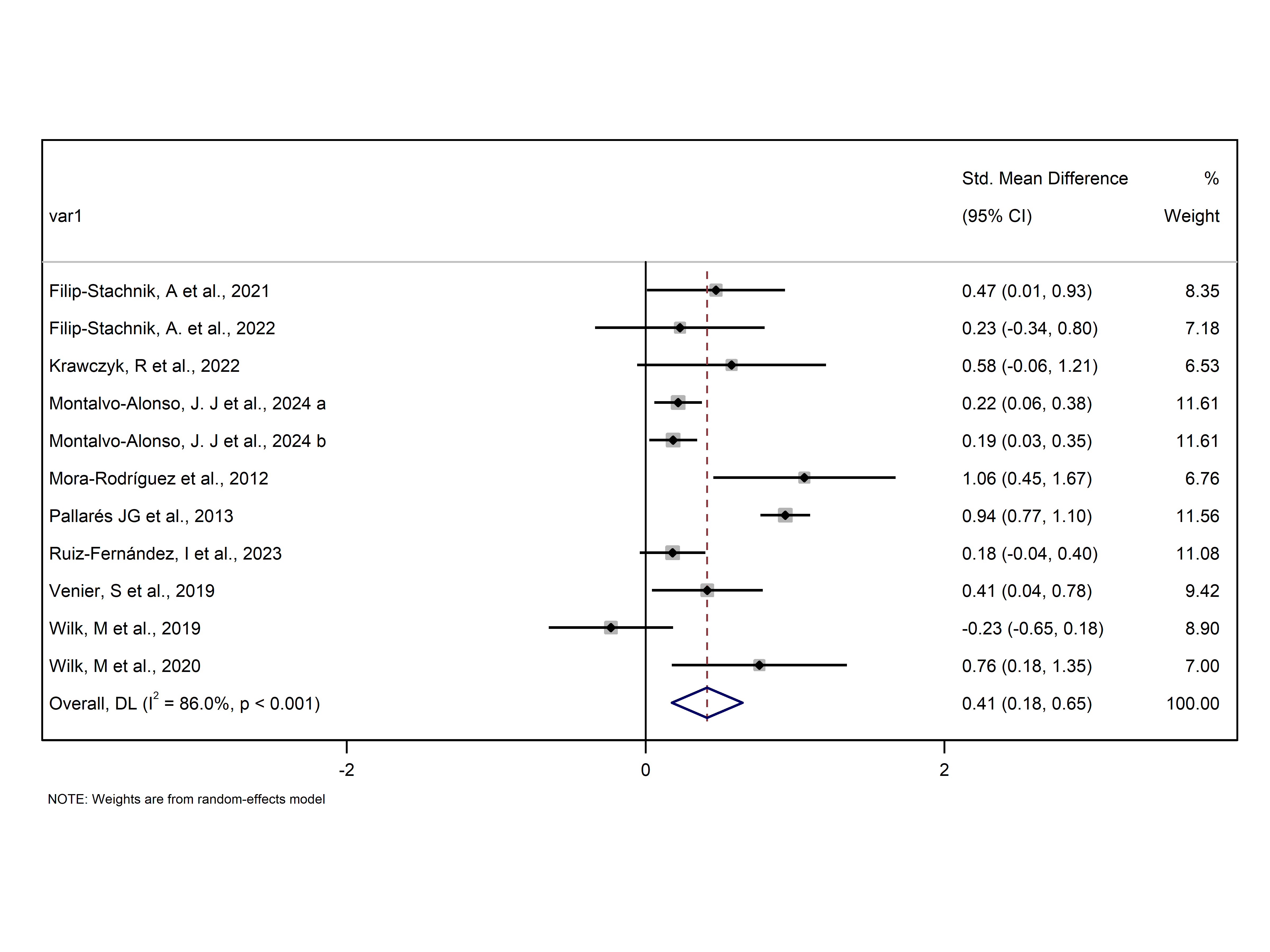

Supplement: Supplementary file 1 [file Data_Sheet_1.ZIP › S14.tif]

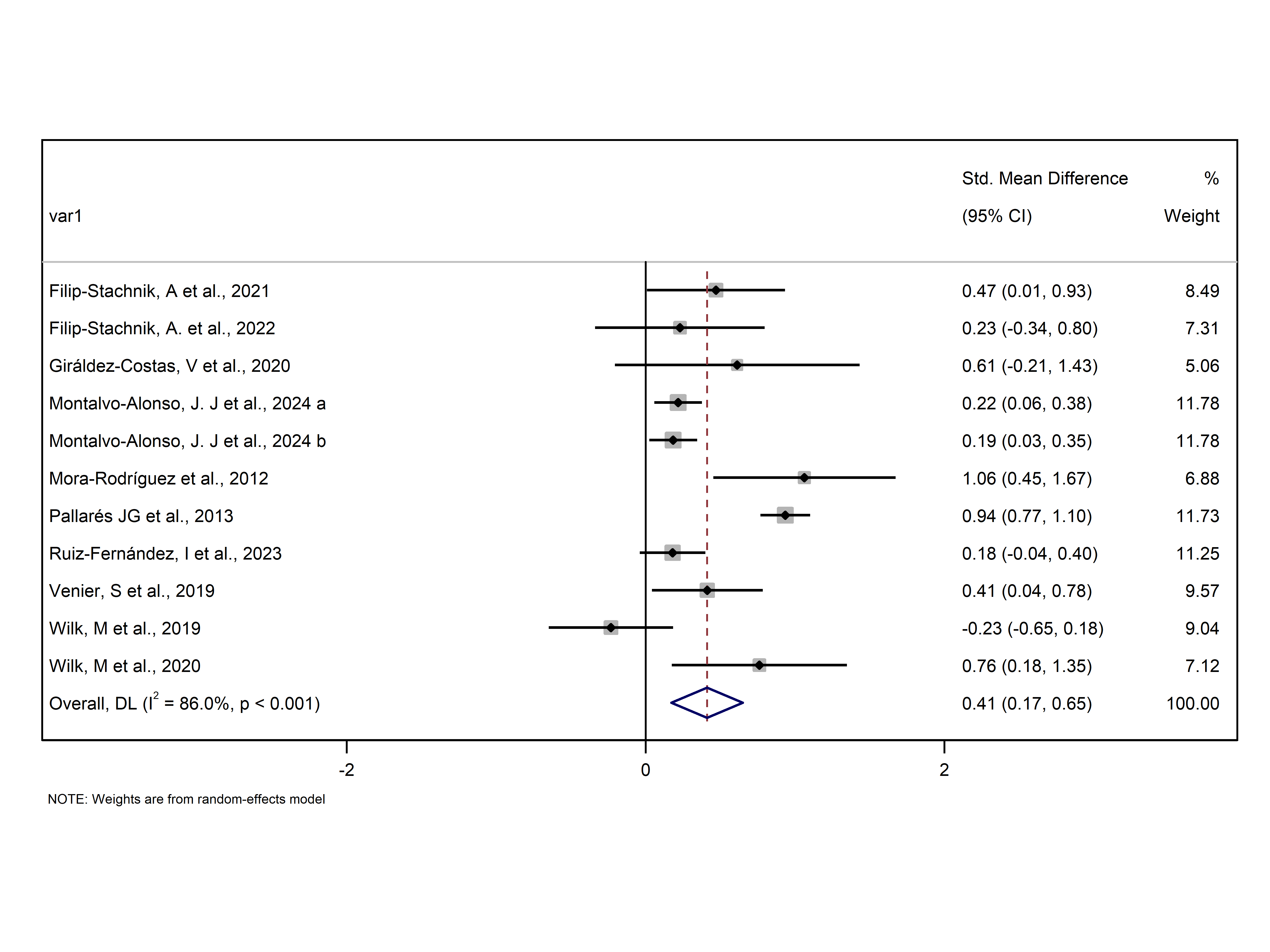

Supplement: Supplementary file 1 [file Data_Sheet_1.ZIP › S15.tif]

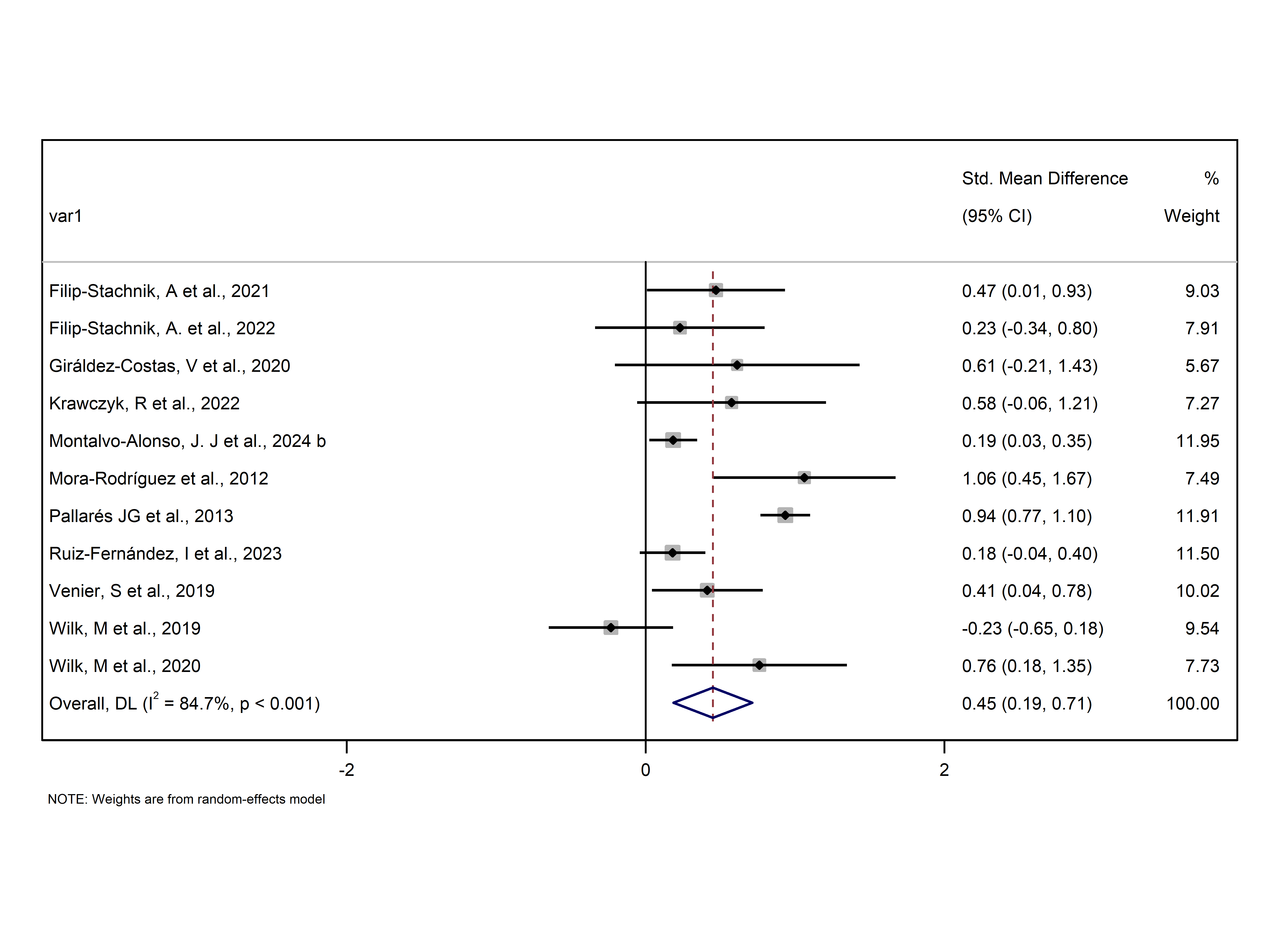

Supplement: Supplementary file 1 [file Data_Sheet_1.ZIP › S16.tif]

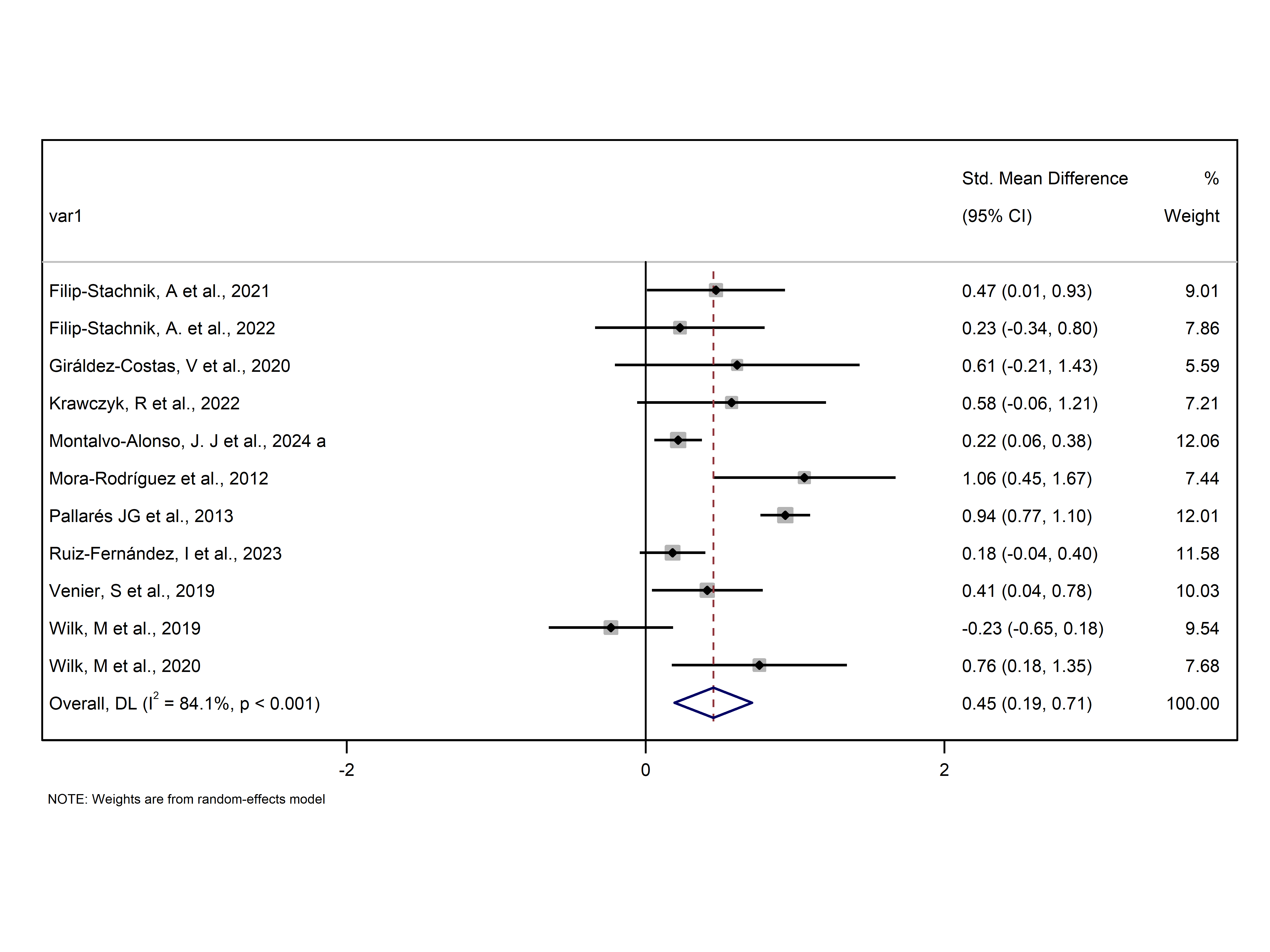

Supplement: Supplementary file 1 [file Data_Sheet_1.ZIP › S17.tif]

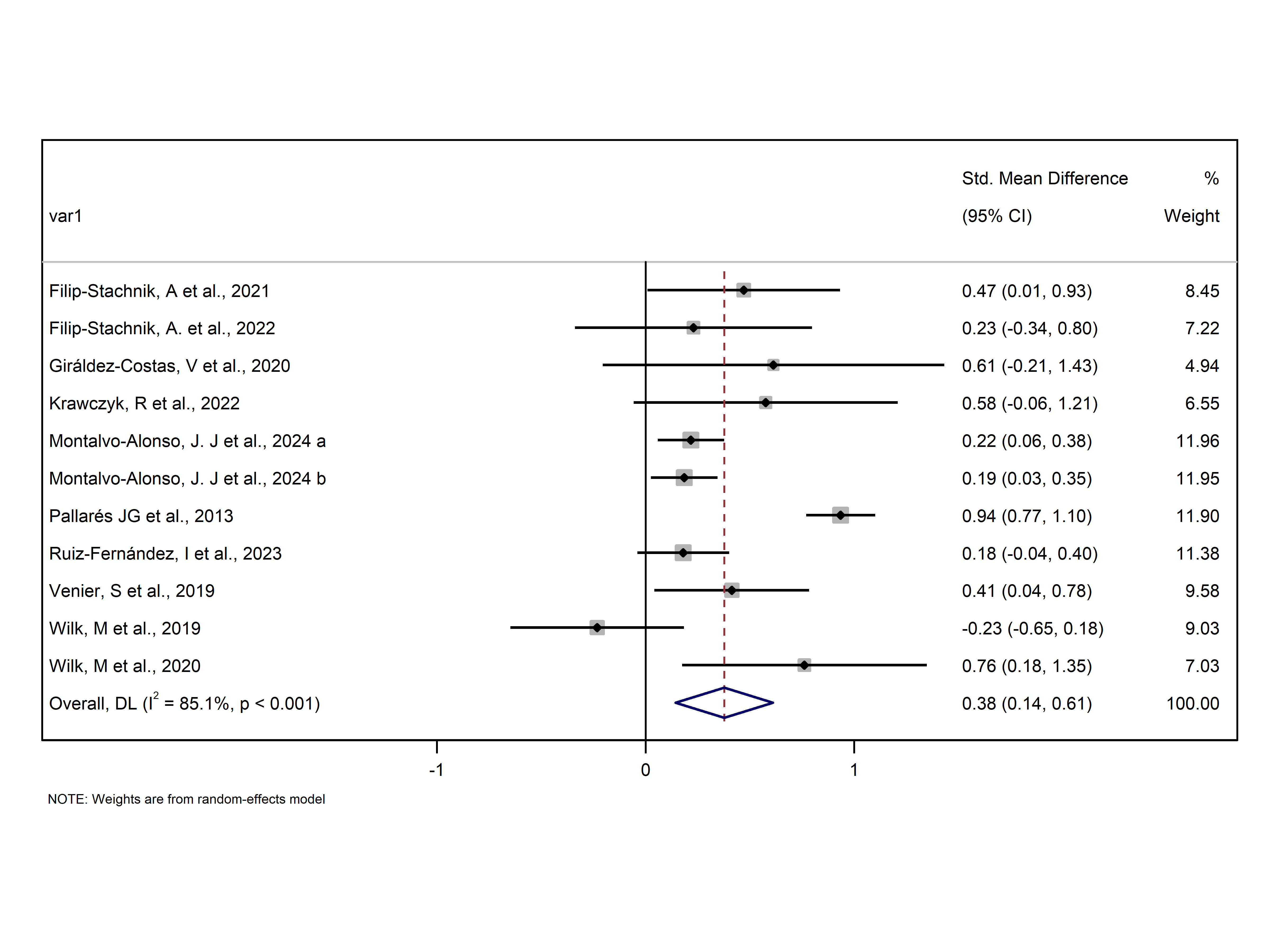

Supplement: Supplementary file 1 [file Data_Sheet_1.ZIP › S18.tif]

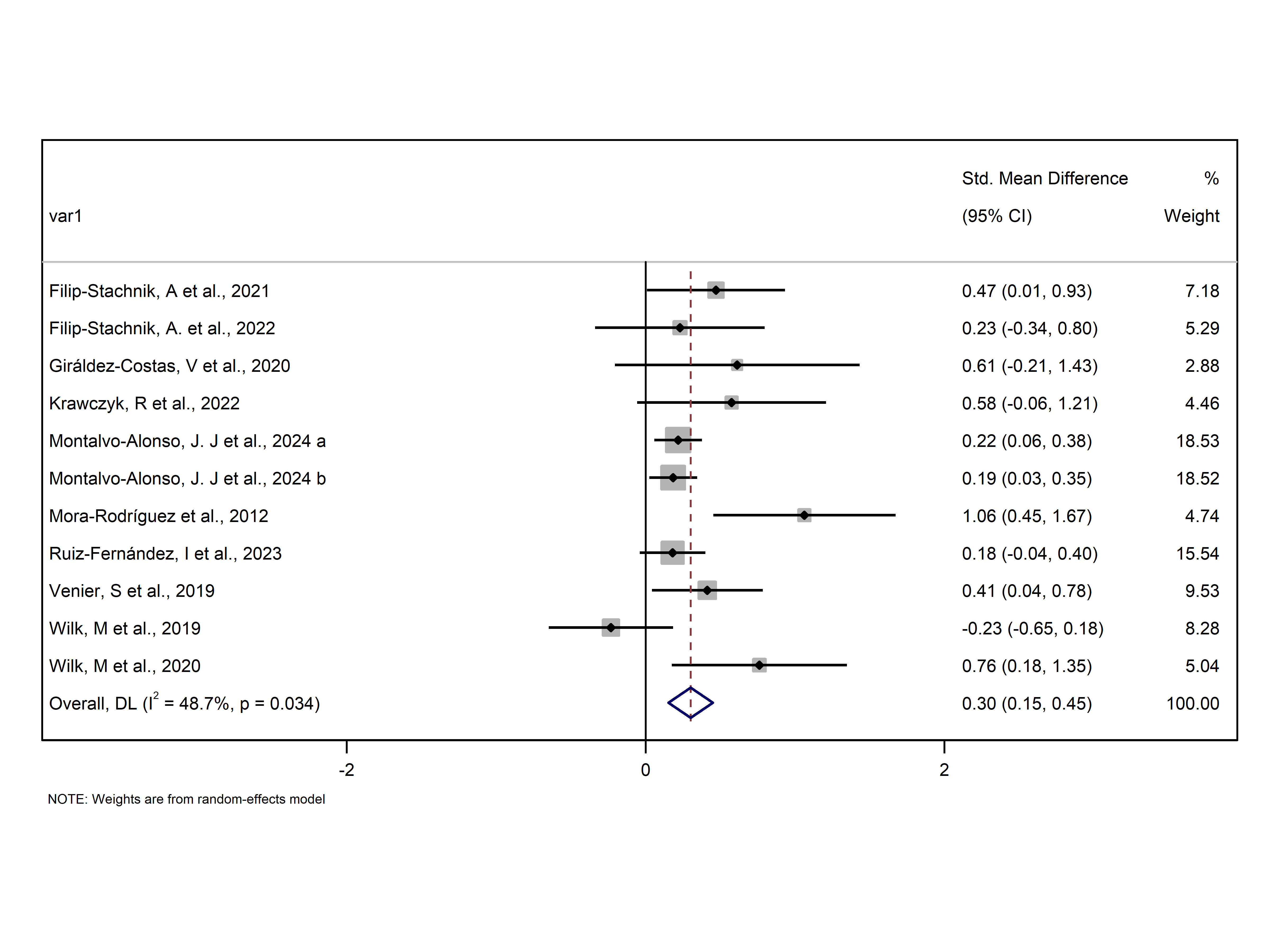

Supplement: Supplementary file 1 [file Data_Sheet_1.ZIP › S19.tif]

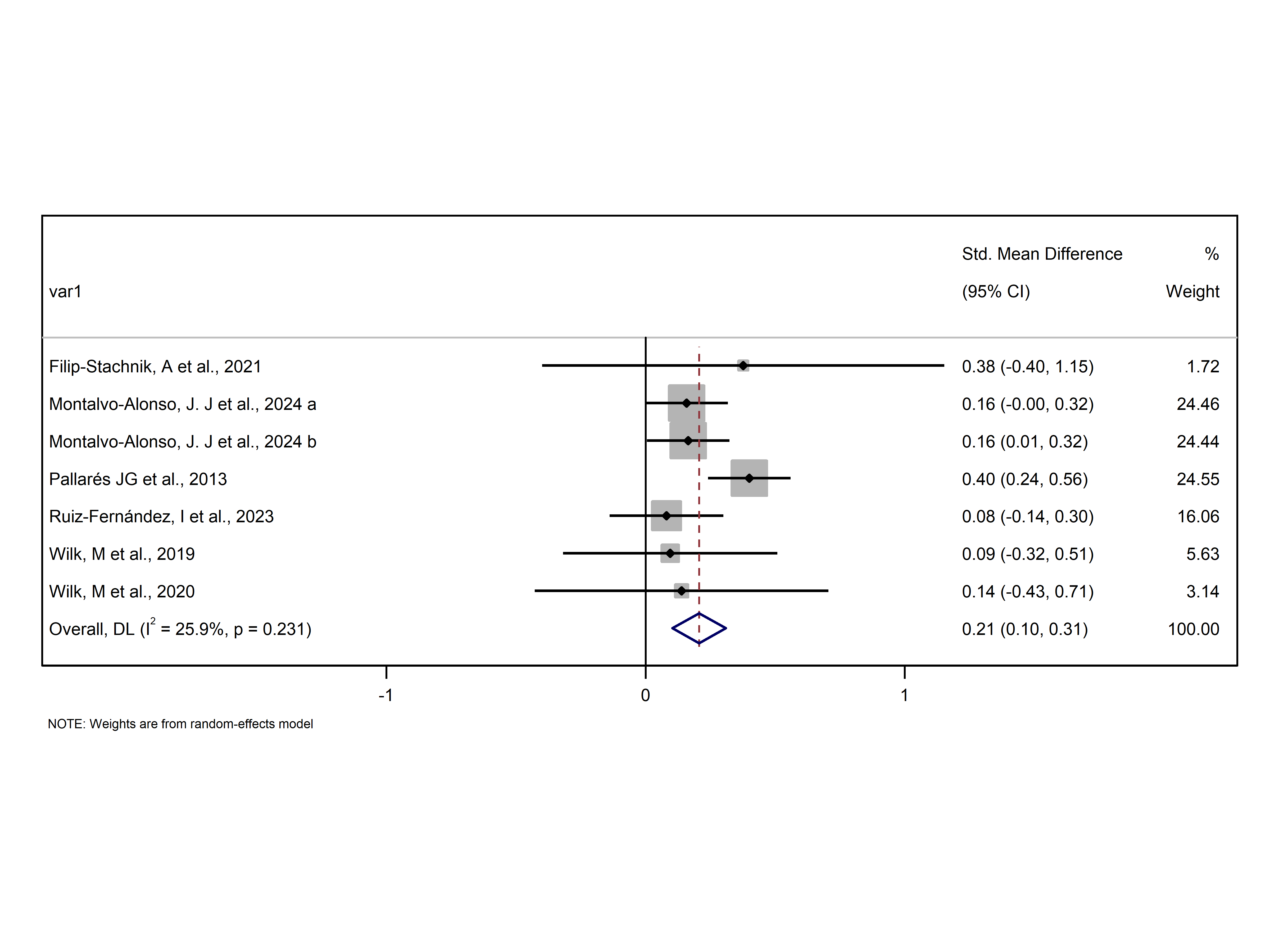

Supplement: Supplementary file 1 [file Data_Sheet_1.ZIP › S2.tif]

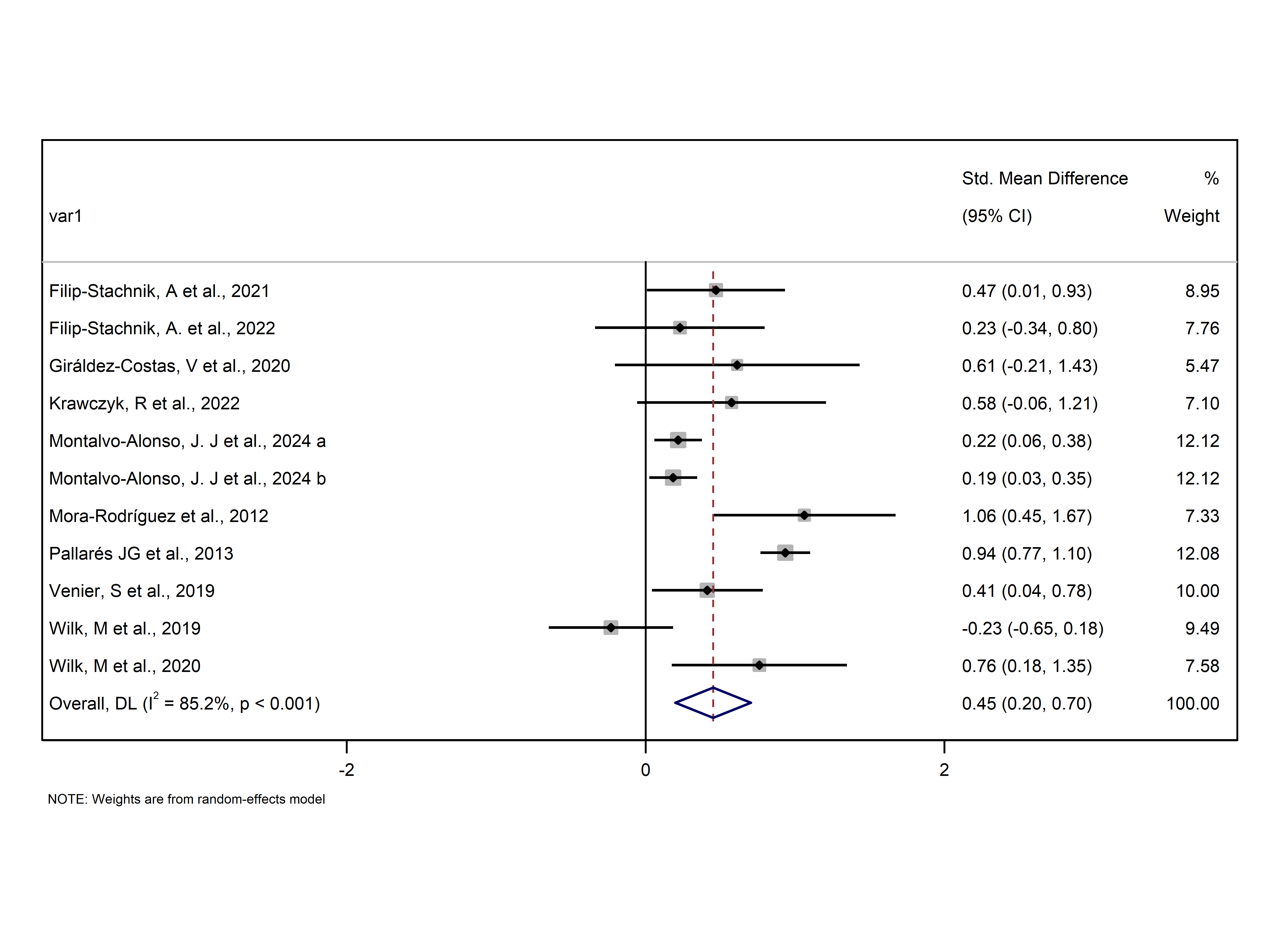

Supplement: Supplementary file 1 [file Data_Sheet_1.ZIP › S20.tif]

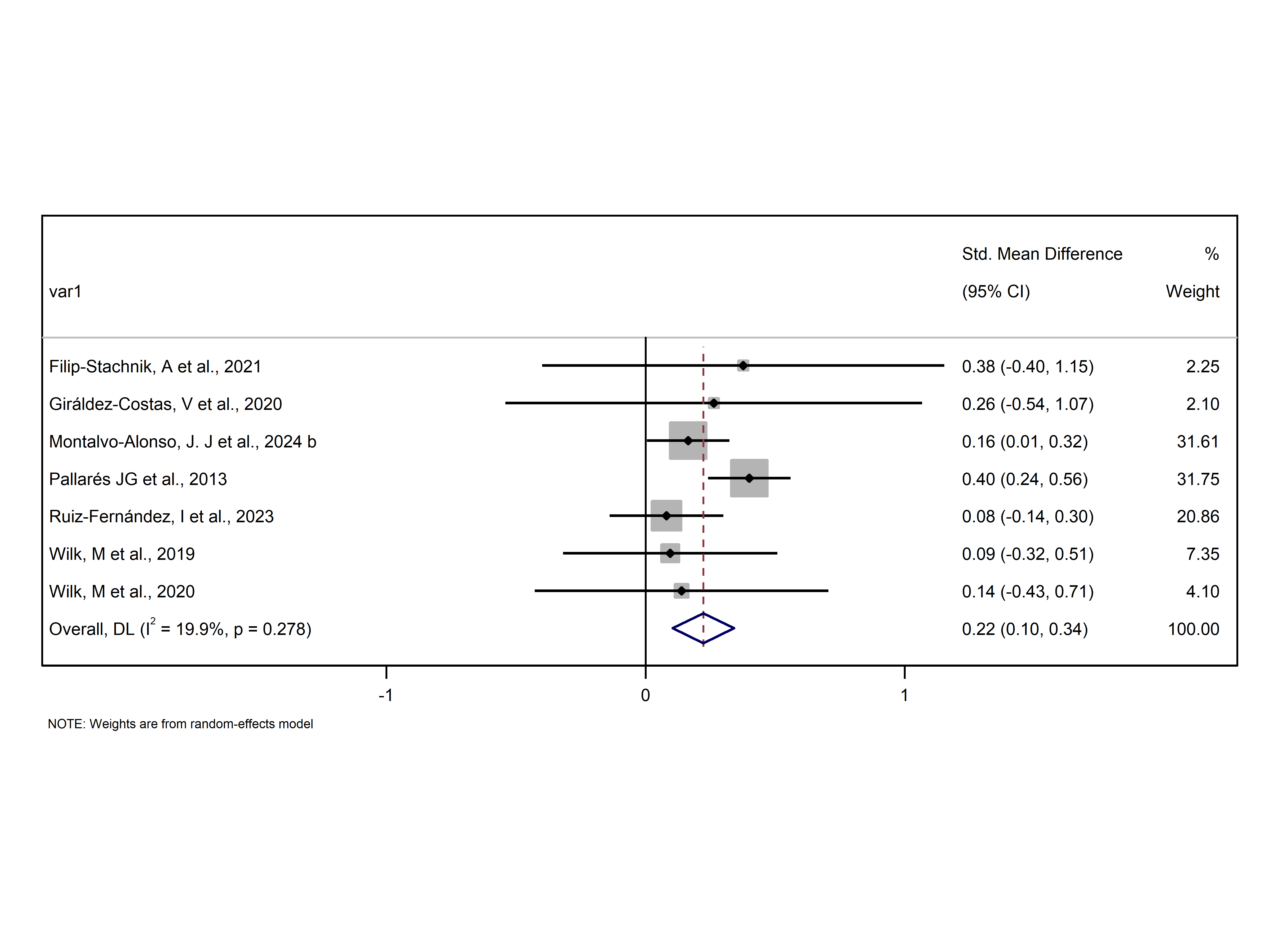

Supplement: Supplementary file 1 [file Data_Sheet_1.ZIP › S3.tif]

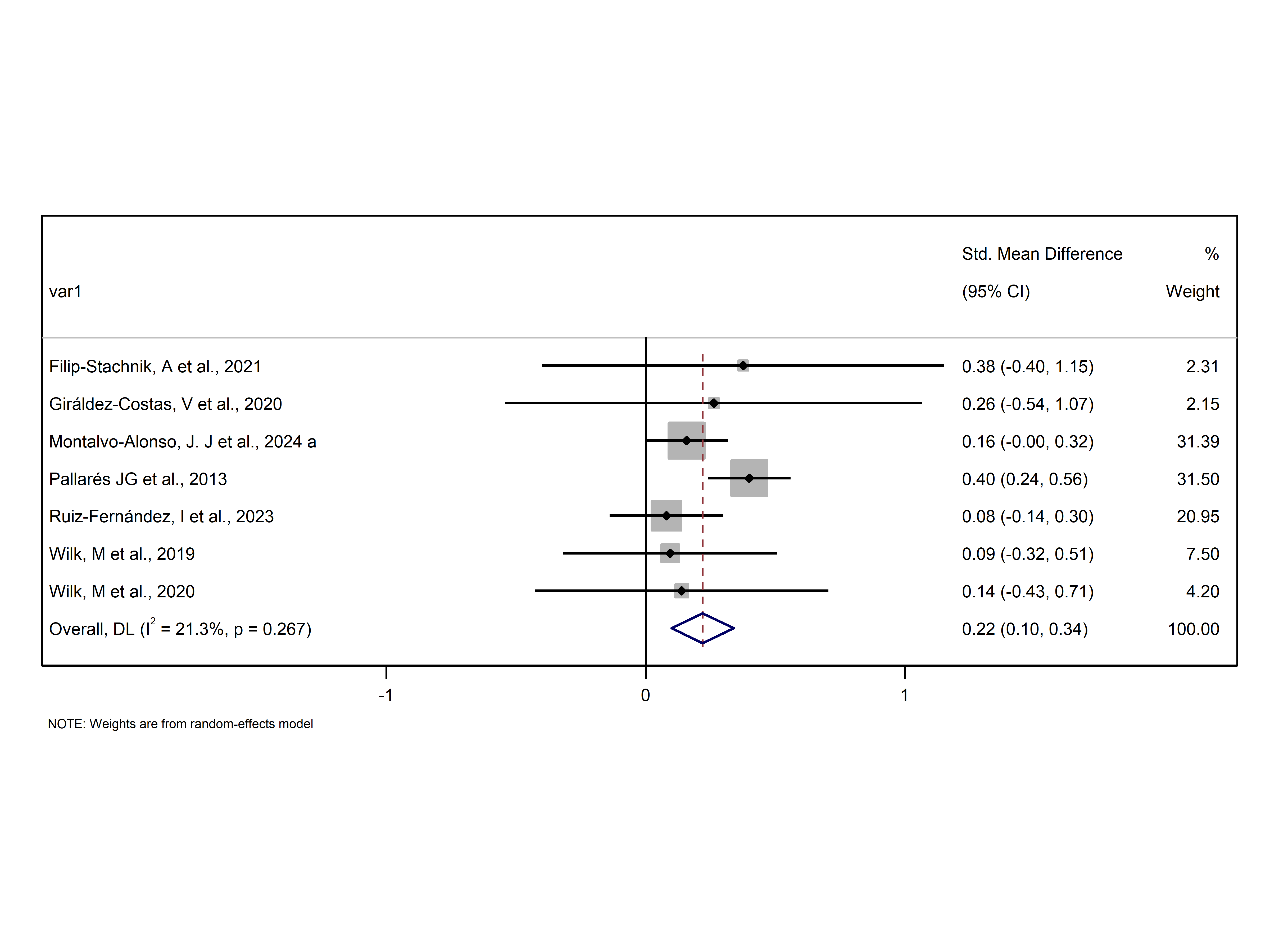

Supplement: Supplementary file 1 [file Data_Sheet_1.ZIP › S4.tif]

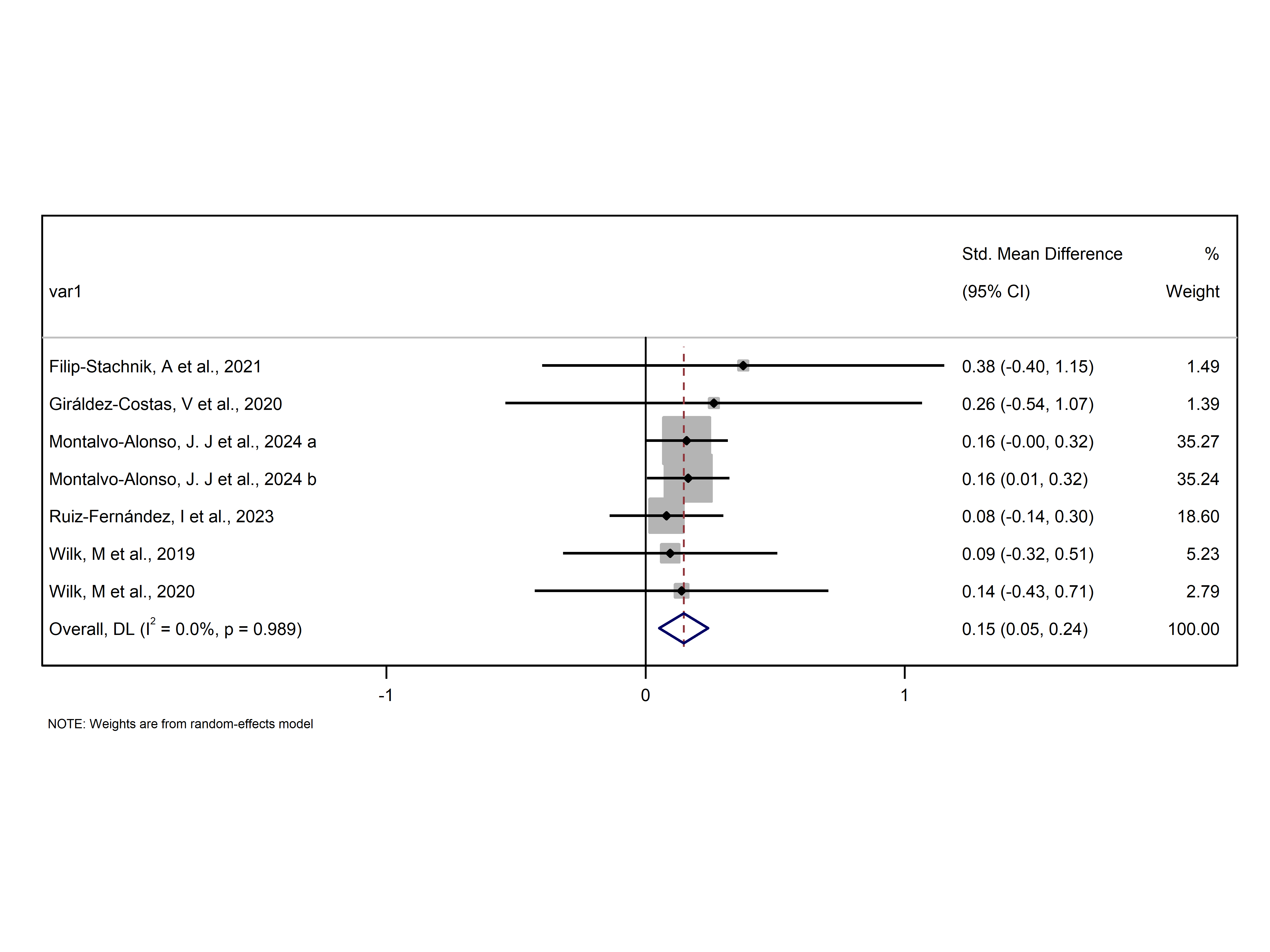

Supplement: Supplementary file 1 [file Data_Sheet_1.ZIP › S5.tif]

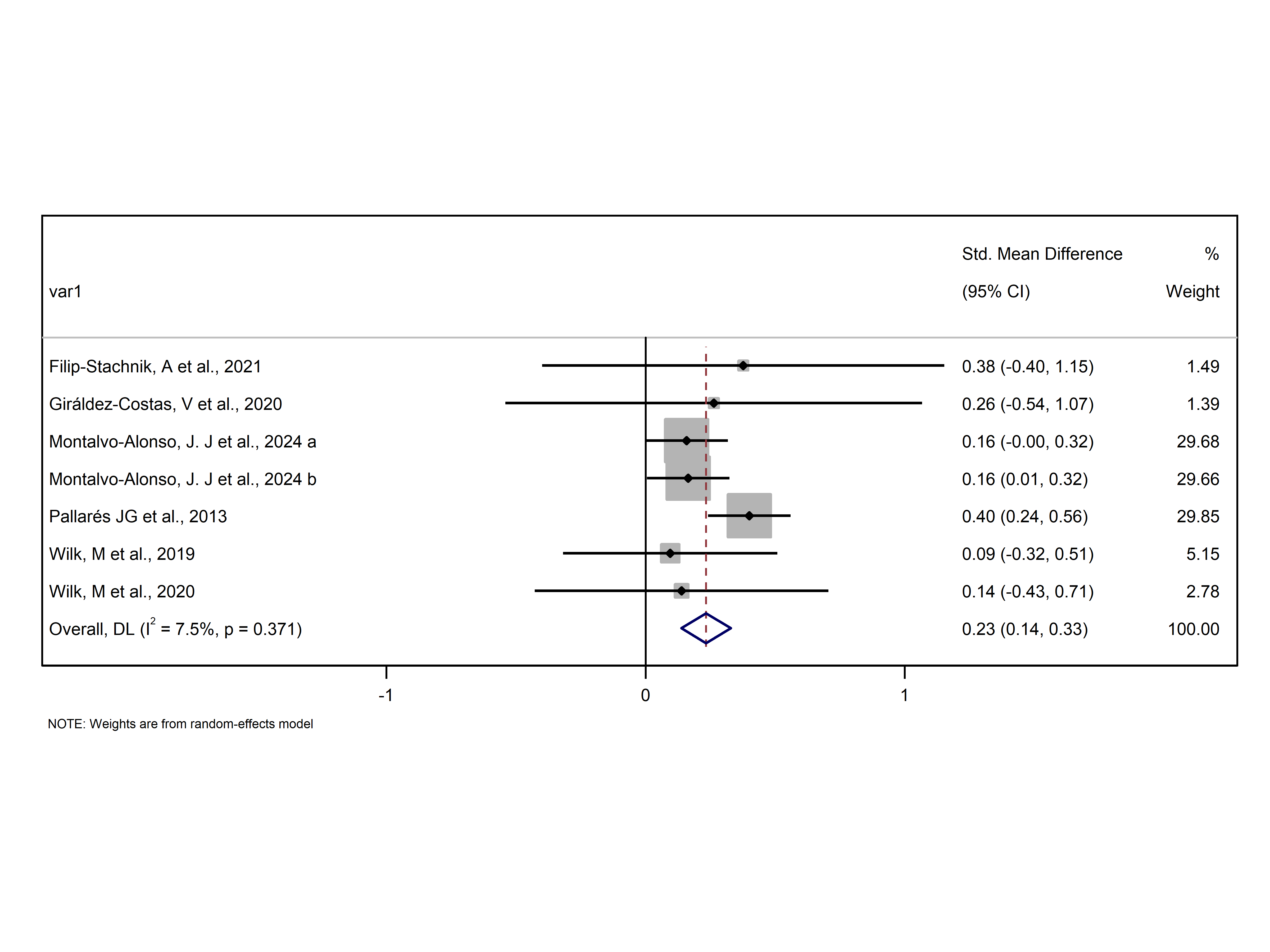

Supplement: Supplementary file 1 [file Data_Sheet_1.ZIP › S6.tif]

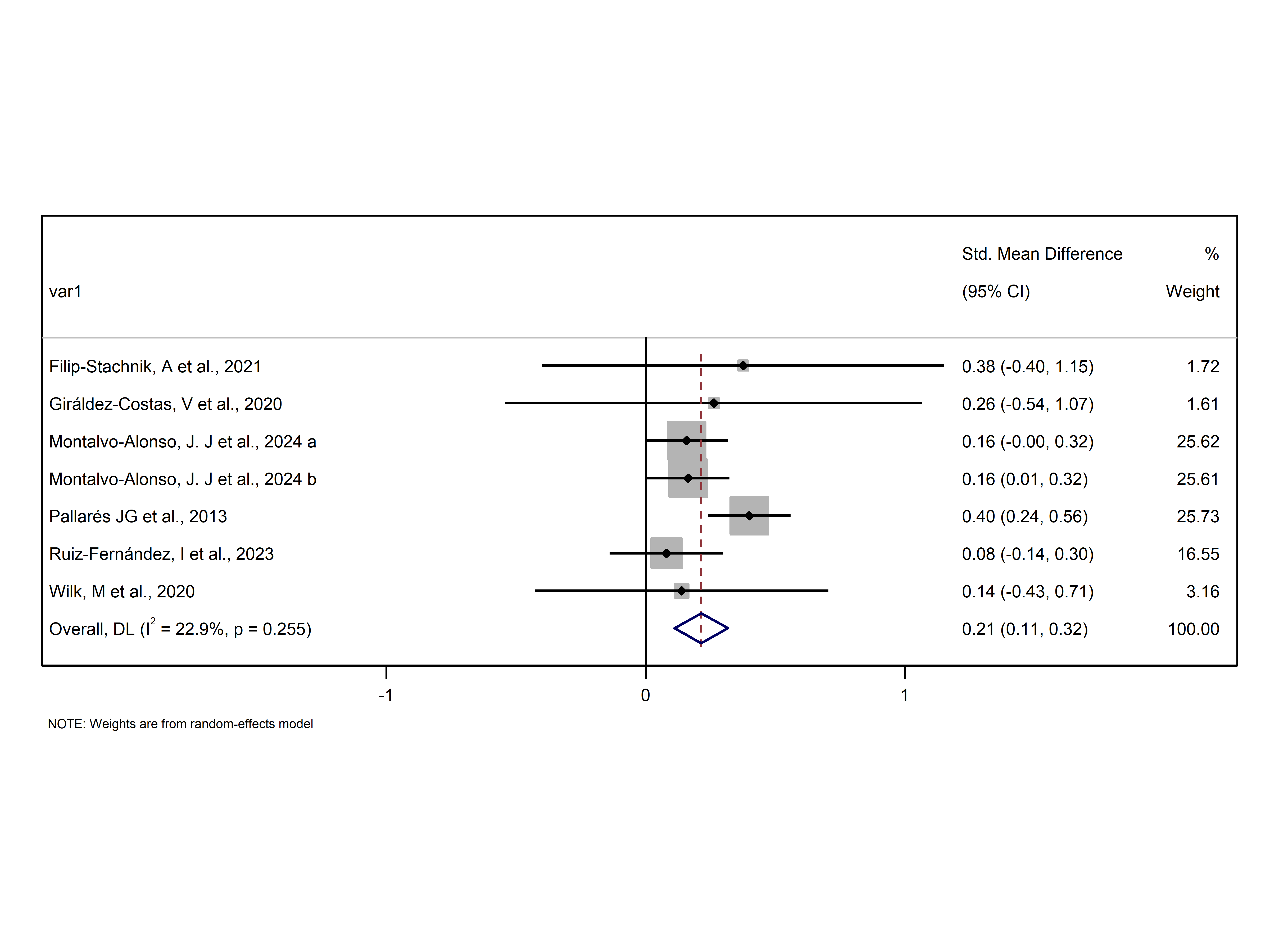

Supplement: Supplementary file 1 [file Data_Sheet_1.ZIP › S7.tif]

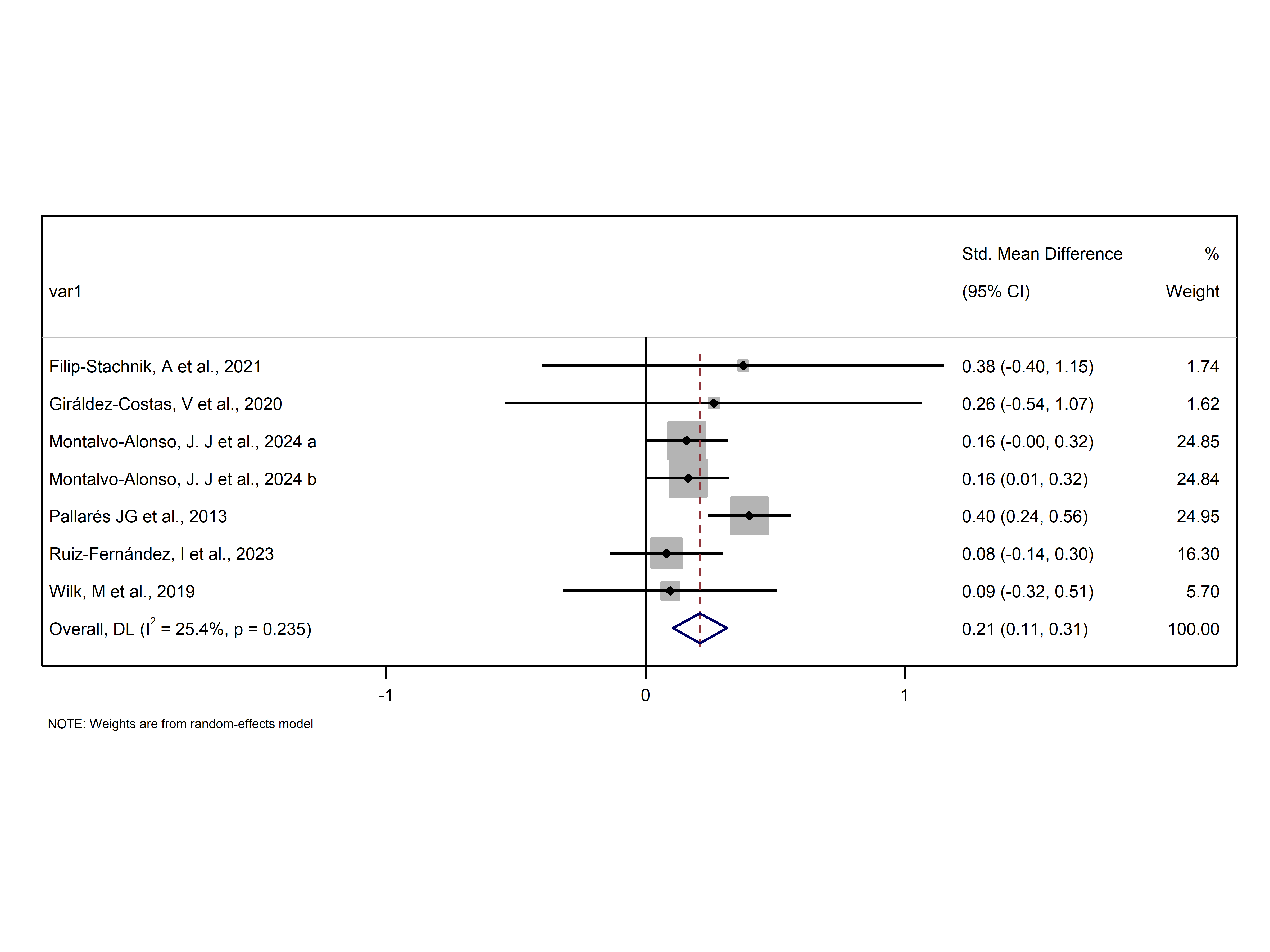

Supplement: Supplementary file 1 [file Data_Sheet_1.ZIP › S8.tif]

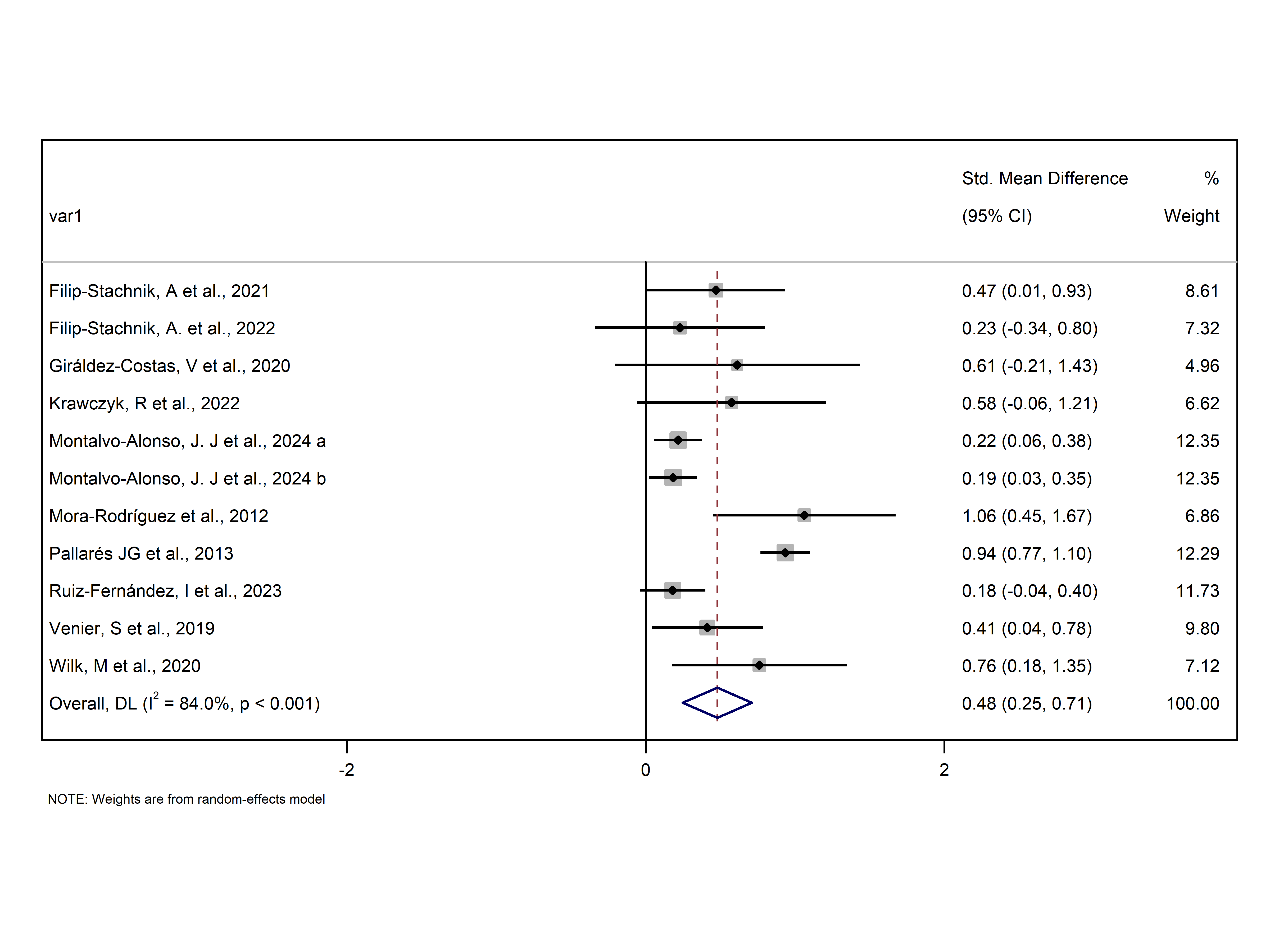

Supplement: Supplementary file 1 [file Data_Sheet_1.ZIP › S9.tif]
