# Supplementary material for: Effects of acute caffeine intake on muscular power during resistance exercise: a systematic review and meta-analysis
Source: Front Nutr. 2025 Oct 7;12:1686283. doi: 10.3389/fnut.2025.1686283 (PMC12537405; doi:10.3389/fnut.2025.1686283)
Supplement: Supplementary file 2 [file Data_Sheet_2.DOCX]

**Stata**

**gen pooled_sd = sqrt(((var4^2 * (var2 - 1) + var7^2 * (var5 - 1)) / (var2 + var5 - 2)))**

**gen smd = (var3 - var6) / pooled_sd**

**gen se_smd = sqrt((1/var2 + 1/var5) + (smd^2 / (2*(var2 + var5))))**

**gen weight = 1 / (se_smd^2)**

**bysort var1: egen sum_weighted_smd = total(smd * weight)**

**bysort var1: egen sum_weights = total(weight)**

**gen weighted_smd = sum_weighted_smd / sum_weights**

**gen combined_se = sqrt(1 / sum_weights)**

**bysort var1: keep if _n == 1**

**drop smd se_smd pooled_sd weight sum_weighted_smd sum_weights var2 var3 var4 var5 var6 var7**

**rename weighted_smd smd**

**rename combined_se se_smd**

**metan smd se_smd, random label(namevar=var1) effect("Std. Mean Difference")**

**graph export "D:/meta/forest_plot.tif", replace width(4800) height(3600)**

**metafunnel smd se_smd**

**graph export "D:/meta/funnel_plot.tif", replace width(4800) height(3600)**

**capture log close**

**log using "D:/meta/egger_results.txt", text replace**

**metabias smd se_smd, egger**

**log close**

**quietly metabias smd se_smd, egger**

**local p_egger = r(p)**

**display "Egger test p-value: " `p_egger'**

**if `p_egger' < 0.05 {**

**display "Egger test significant (p < 0.05), performing trim-and-fill analysis"**

**metatrim smd se_smd, reffect**

**graph export "D:/meta/trim_and_fill_plot.tif", replace width(4800) height(3600)**

**capture log close**

**log using "D:/meta/trim_and_fill_results.txt", text replace**

**metatrim smd se_smd, reffect**

**log close**

**}**

**else {**

**display "Egger test not significant (p >= 0.05), no trim-and-fill analysis needed"**

**}**

**capture log close**

**log using "D:/meta/sensitivity_analysis.txt", text replace**

**gen study_id = _n**

**local total_studies = _N**

**preserve**

**forvalues id = 1/`total_studies' {**

**restore, preserve**

**keep if study_id != `id'**

**capture {**

**quietly metan smd se_smd, random nograph**

**local effect = r(ES)**

**local lower_ci = r(ci_low)**

**local upper_ci = r(ci_upp)**

**local p_value = r(p_z)**

**capture log using "D:/meta/sensitivity_analysis.txt", text append**

**display "Excluding study ID `id': ES = `effect', 95% CI [`lower_ci', `upper_ci'], p = `p_value'"**

**log close**

**metan smd se_smd, random label(namevar=var1) effect("Std. Mean Difference")**

**local filename = "D:/meta/sensitivity_excluding_study_`id'.tif"**

**graph export "`filename'", replace width(4800) height(3600)**

**}**

**}**

**restore**

**capture log close**

**R tool

Risk assessment**library(readxl)

library(dplyr)

library(tidyr)

library(ggplot2)

library(scales)

library(robvis)

save_path <- "C:/Users"

dir.create(save_path, recursive = TRUE, showWarnings = FALSE)

data <- read_excel("C:/Users /data.xlsx")

data_rob <- data

data_rob[data_rob == "Low"] <- "Low risk"

data_rob$Weights <- 1

data_rob$`Overall risk of bias` <- data_rob$Overall

domains <- c("D1","D2","D3","D4","D5","Overall risk of bias")

long <- data_rob %>%

select(all_of(domains)) %>%

pivot_longer(cols = everything(), names_to = "Domain", values_to = "Rating")

long$Rating <- factor(long$Rating, levels = c("Low risk", "Some concerns", "High risk"))

summary_df <- long %>%

group_by(Domain, Rating) %>%

summarise(n = n(), .groups = "drop") %>%

group_by(Domain) %>%

mutate(prop = n / sum(n)) %>%

ungroup()

domain_labels <- c(

"D1" = "Bias arising from the randomization process",

"D2" = "Bias due to deviations from intended interventions",

"D3" = "Bias due to missing outcome data",

"D4" = "Bias in measurement of the outcome",

"D5" = "Bias in selection of the reported result",

"Overall risk of bias" = "Overall risk of bias"

)

summary_df$Domain <- factor(summary_df$Domain, levels = rev(domains))

cols <- c("Low risk" = "#02C100",

"Some concerns" = "#E2DF07",

"High risk" = "#BF0000")

p_summary <- ggplot(summary_df, aes(x = prop, y = Domain, fill = Rating)) +

geom_bar(stat = "identity", width = 0.65, color = "black") +

scale_x_continuous(labels = percent_format(accuracy = 1),

limits = c(0,1.05), breaks = seq(0,1,0.25),

expand = c(0,0)) +

scale_fill_manual(values = cols, na.value = "grey80") +

scale_y_discrete(labels = domain_labels) +

labs(x = NULL, y = NULL, fill = "") +

theme_minimal(base_size = 12) +

theme(

text = element_text(family = "Times New Roman", size = 12),

panel.grid.major.y = element_blank(),

axis.text.y = element_text(face = "bold"),

legend.position = "bottom"

)

ggsave(filename = file.path(save_path, "risk_of_bias_summary_full.pdf"),

plot = p_summary, width = 10, height = 4, device = cairo_pdf)

1. **traffic light**

library(ggplot2)

library(dplyr)

library(tidyr)

library(readxl)

data <- read_excel("C:/Users/data.xlsx")

data_long <- data %>%

pivot_longer(cols = c(D1, D2, D3, D4, D5, Overall),

names_to = "Domain",

values_to = "Risk") %>%

mutate(

Risk = factor(Risk, levels = c("Low", "Some concerns")),

# 创建符号列：Low用"+", Some concerns用"-"

Symbol = ifelse(Risk == "Low", "+", "-")

)

domain_labels <- c(

"D1" = "D1: Randomization bias",

"D2" = "D2: Deviations bias",

"D3" = "D3: Missing data bias",

"D4" = "D4: Measurement bias",

"D5" = "D5: Selection bias",

"Overall" = "Overall risk"

)

rob_traffic_light <- ggplot(data_long, aes(x = Domain, y = Study)) +

geom_point(aes(fill = Risk), shape = 21, size = 10, color = "black") +

geom_text(aes(label = Symbol), size = 5, color = "black", fontface = "bold") +

scale_fill_manual(values = c("Low" = "green", "Some concerns" = "yellow")) +

scale_x_discrete(labels = domain_labels) +

theme_minimal() +

theme(

axis.text.x = element_text(angle = 0, hjust = 0.5, size = 10),

axis.text.y = element_text(size = 9),

panel.grid = element_blank(),

legend.position = "bottom",

plot.title = element_text(hjust = 0.5, face = "bold"),

plot.caption = element_text(hjust = 0, size = 9, face = "plain",

margin = margin(t = 10)),

plot.margin = margin(1, 1, 1, 1, "cm"),

axis.title.x = element_text(margin = margin(t = 10)),

axis.title.y = element_text(margin = margin(r = 10))

) +

labs(

title = "Risk of Bias Assessment",

x = "Domain",

y = "Study",

fill = "Risk Level",

caption = "Domain definitions:

D1: Bias arising from the randomization process

D2: Bias due to deviations from intended interventions

D3: Bias due to missing outcome data

D4: Bias in measurement of the outcome

D5: Bias in selection of the reported result

Overall: Overall risk of bias"

) +

scale_x_discrete(expand = c(0.05, 0.05)) +

scale_y_discrete(expand = c(0.05, 0.05))

print(rob_traffic_light)

# 保存为PDF

save_path <- "C:/Users/ /risk_of_bias_plot.pdf"

ggsave(save_path, rob_traffic_light, width = 10, height = 8, device = "pdf")

cat("图形已保存至:", save_path)

1. **subgroup**

rm (list = ls())

library(forestploter)

library(grid)

library(readxl)

mydata1 <- read_excel("mydata1.xlsx")

View(mydata1)

mydata1 <- as.data.frame(apply(mydata1, 2, function(x) ifelse(is.na(x), "", x)))

mydata1$`Forest plot (REM)` <- paste(rep(" ", 10), collapse = " ")

tm <- forest_theme(base_size = 10,

refline_gp = gpar(col = "black"))

col8_data <- unlist(mydata1[, 8])

mydata1[, 8] <- ifelse(col8_data != "", paste0(as.numeric(col8_data) * 100, "%"), col8_data)

mydata1$SMD <- as.numeric(mydata1$SMD)

mydata1$lower.95 <- as.numeric(mydata1$lower.95)

mydata1$upper.95 <- as.numeric(mydata1$upper.95)

p <- forest(mydata1[,c(1,2,10,6,7,8,9)],

est = mydata1$SMD,

lower = mydata1$lower.95,

upper = mydata1$upper.95,

#sizes = 0.8,

ci_column = 3,

ref_line = 0,

arrow_lab = c("Favours Control", "Favours Experimental"),

#xlim = c(-25, 25),

ticks_at = c(-0.9,-0.6,-0.3,0, 0.3, 0.6, 0.9),

#footnote = "Pairwise meta-analyses of cardiometabolic health.",

theme = tm)

p <- add_border(p, part = "header", where = "bottom")

p <- add_border(p, part = "header", where = "top")

print(p)
