# Supplementary material for: Effects of acute caffeine intake on muscular power during resistance exercise: a systematic review and meta-analysis
Source: Front Nutr. 2025 Oct 7;12:1686283. doi: 10.3389/fnut.2025.1686283 (PMC12537405; doi:10.3389/fnut.2025.1686283)
Supplement: Supplementary file 3 [file Data_Sheet_3.DOCX]

**Search strategy**

|  | **Terms** | **Search Strategy** |
| --- | --- | --- |
| #1 | Caffeine | "Caffeine"[MeSH Terms] OR "Caffeine"[Title/Abstract] OR "coffee"[Title/Abstract] OR "caffeinated"[Title/Abstract] OR "1 3 7 trimethylxanthine"[Title/Abstract] OR "Vivarin"[Title/Abstract] OR "Caffedrine"[Title/Abstract] OR "coffeinum n"[Title/Abstract] OR "Dexitac"[Title/Abstract] OR "no doz"[Title/Abstract] OR "Quick-Pep"[Title/Abstract] |
| #2 | Resistance Training | "Resistance Training"[MeSH Terms] OR "resistance exercise"[Title/Abstract] OR "Resistance Training"[Title/Abstract] OR "mean velocity"[Title/Abstract] OR "peak velocity"[Title/Abstract] OR "strength exercise"[Title/Abstract] OR "strength training"[Title/Abstract] OR "bench press"[Title/Abstract] OR "Speed"[Title/Abstract] OR "mean power"[Title/Abstract] OR "peak power"[Title/Abstract] OR "Squat"[Title/Abstract] OR "leg press"[Title/Abstract] OR "leg extension"[Title/Abstract] OR "ballistic"[Title/Abstract] |
| #3 | #1 AND #2 |  |
